# Functional connectivity interacts with visual perceptual learning for visual field recovery in chronic stroke

**DOI:** 10.1038/s41598-024-52778-x

**Published:** 2024-02-08

**Authors:** Eun Namgung, Yong-Hwan Kim, Eun-Jae Lee, Yuka Sasaki, Takeo Watanabe, Dong-Wha Kang

**Affiliations:** 1 Asan Institute for Life Sciences, Asan Medical Center, Seoul, South Korea; 2 Nunaps Inc., Seoul, South Korea; 3 Department of Cognitive, Linguistic and Psychological Sciences, Brown University, Providence, USA; 4 Department of Neurology, Asan Medical Center, University of Ulsan College of Medicine, Seoul, South Korea

**Keywords:** Neuroscience, Neurology

## Abstract

A reciprocal relationship between perceptual learning and functional brain changes towards perceptual learning effectiveness has been demonstrated previously; however, the underlying neural correlates remain unclear. Further, visual perceptual learning (VPL) is implicated in visual field defect (VFD) recovery following chronic stroke. We investigated resting-state functional connectivity (RSFC) in the visual cortices associated with mean total deviation (MTD) scores for VPL-induced VFD recovery in chronic stroke. Patients with VFD due to chronic ischemic stroke in the visual cortex received 24 VPL training sessions over 2 months, which is a dual discrimination task of orientation and letters. At baseline and two months later, the RSFC in the ipsilesional, interhemispheric, and contralesional visual cortices and MTD scores in the affected hemi-field were assessed. Interhemispheric visual RSFC at baseline showed the strongest correlation with MTD scores post-2-month VPL training. Notably, only the subgroup with high baseline interhemispheric visual RSFC showed significant VFD improvement following the VPL training. The interactions between the interhemispheric visual RSFC at baseline and VPL led to improvement in MTD scores and largely influenced the degree of VFD recovery. The interhemispheric visual RSFC at baseline could be a promising brain biomarker for the effectiveness of VPL-induced VFD recovery.

## Introduction

Visual field defect (VFD), a debilitating complication after a posterior circulation stroke, significantly reduces overall quality of life of stroke patients, including mood, cognition, and daily functioning^1^. Despite the clinical importance of VFD, rehabilitation strategies for improving VFD are still being developed^2–4^. Visual perceptual learning (VPL), defined as the training-dependent sustained improvement in visual-perception performance^5–8^, is gaining attention as a potential intervention for VFD following posterior circulation stroke^9,10^. Specifically, diverse types of visual discrimination or detection (i.e. flickering light/letters, orientation, motion, grating, and shapes of visual stimuli presented repetitively in blind or normal visual fields) trainings have been applied for visual field recovery in chronic occipital stroke, leading to the development of promising digital therapeutics^9,11–16^. Controversy surrounds the efficacy of vision restoration, with ongoing debates on the optimal VPL protocol concerning detection versus discrimination, selection of visual stimuli types, as well as visual presentation in normal versus blind fields^14,16^.

Recent research posits that VPL interacts with the functional neural circuits underlying sensory perception and learning, influencing the effectiveness of VPL^8,17–20^. However, despite accumulating evidence, the neural correlates of this interrelationships and their impact on perceptual learning remain inconclusive. Human functional magnetic resonance imaging (fMRI) studies indicate a reciprocal relationship between VPL and visual functional connectivity towards VFD recovery in stroke. Specifically, functional connectivity of the visual cortex is associated with perceptual learning performance^8,17–20^, with the visual cortex showing functional alterations as an adaptation to stroke lesions in relation to VFD recovery after perceptual learning^21^.

Accumulating evidence suggests that the baseline functional connectivity within the visual cortex, which has predicted perceptual learning performance^22^, may play a pivotal role in VPL-induced VFD recovery after stroke. This may be due to its ability to influence the degree of VFD-induced recovery through VPL interaction. For instance, interhemispheric functional connectivity, shifting from the contralesional to ipsilesional, predicted post-stroke performance including spontaneous VFD recovery in acute stroke^23–25^. However, whether baseline functional connectivity within the visual cortex is directly related to VFD recovery through VPL requires elucidation, considering its clinical significance as a predictive brain biomarker. Establishing this association can facilitate the development of tailored VPL protocols.

**Figure 1:**
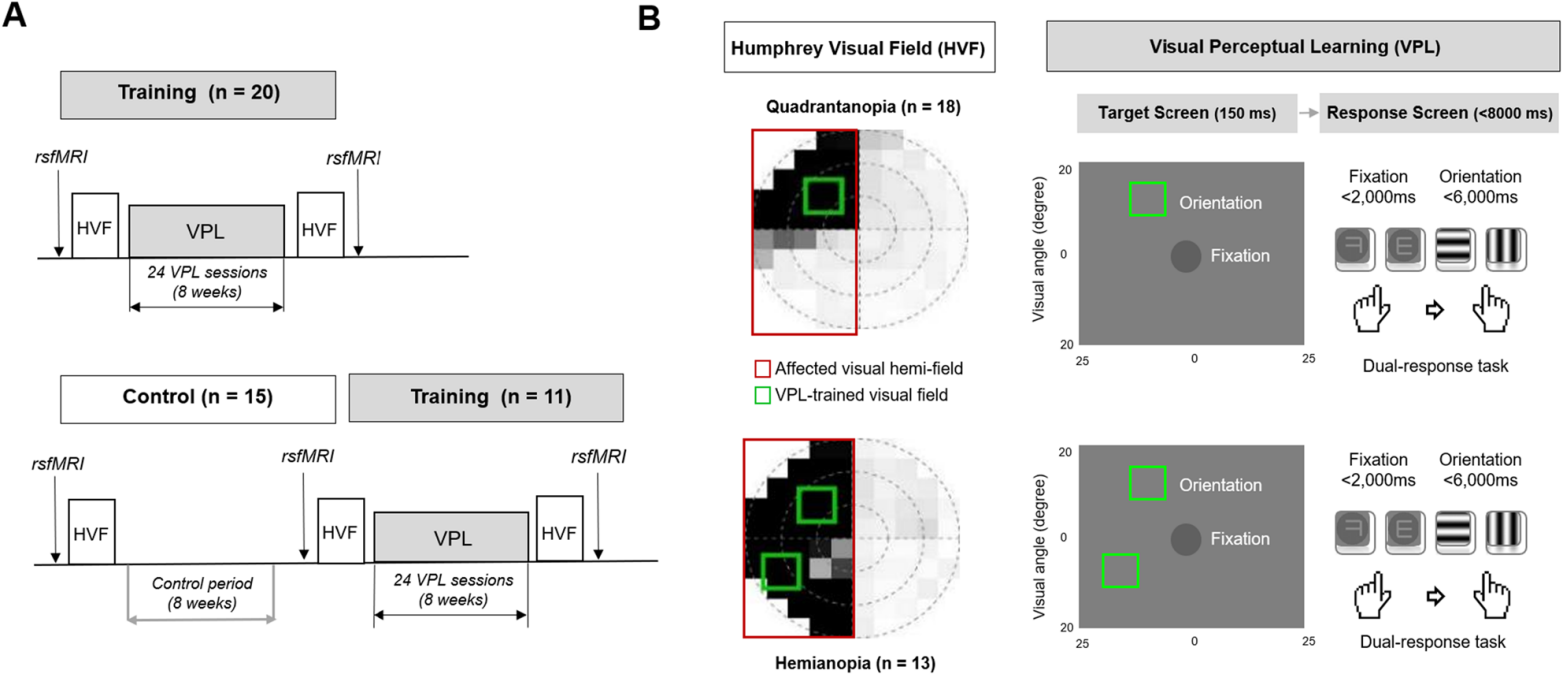
Study design and visual perceptual learning (**A**) The final analysis included patients with VFD following chronic ischemic stroke who received 24 sessions of VPL training for 2 months (VPL training group, n = 31) and those who underwent no-training for a control period of 2 months (control group, n = 15). Humphrey Visual Field (HVF) tests and resting-state functional magnetic resonance imaging were performed before and after the 2-month period. (**B**) The mean total deviation scores in the affected visual hemi-field (red box) were calculated using the HVF tests. During VPL, patients were asked to identify the fixation letter on the center (“ᄏ” or “ᄐ” in Korean) and the direction of the orientation of grating (horizontal or vertical) of the text square by pressing two of the four response buttons, while fixating on the center of the screen. The training location (10° × 10°, green box) was primarily centered 10 degrees away from the fixation point at the center of the display, but it was adjusted towards the peripheral side if the VFD predominantly affected the edge of the affected hemifield, as determined by the baseline HVF test. The orientation stimuli were randomly presented in a random location for the normal quadrant or a fixed location for the individualized defective quadrant of the visual field based on baseline HVF test results. A single location training of either upper or lower quadrant was provided for patients with quadrantanopia, while the two location training of both upper and lower quadrants was provided for patients with hemianopia. HVF, Humphrey Visual Field Test; rsfMRI, resting-state functional magnetic resonance imaging; VPL, visual perceptual learning.

Here, we aimed to assess whether baseline resting-state functional connectivity (RSFC) patterns in the ipsilesional, interhemispheric, and contralesional visual regions predict VPL-induced VFD recovery in patients who experienced chronic ischemic stroke (Fig. 1). The current study investigated the neural plasticity, neurobehavioral associations, and potential predictive brain biomarkers that underpin VPL-induced VFD recovery in chronic stroke. Given the central role of the interhemispheric occipital RSFC in spontaneous VFD recovery after acute stroke^25^, we hypothesized that baseline RSFC within the interhemispheric visual regions may be related to the degree of later VPL-induced VFD recovery in chronic ischemic stroke. The identification of brain biomarkers for the the effectiveness of VPL-induced VFD recovery may facilitate the development of individualized VPL protocols.

## Results

### Neurobehavioral associations: associations between visual RSFC and visual performance in relation to VPL

To investigate changes in neurobehavioral associations after VPL, associations between visual RSFC and mean total deviation (MTD) score were investigated at pre-VPL and post-VPL, respectively, in the training group.

**Figure 2:**
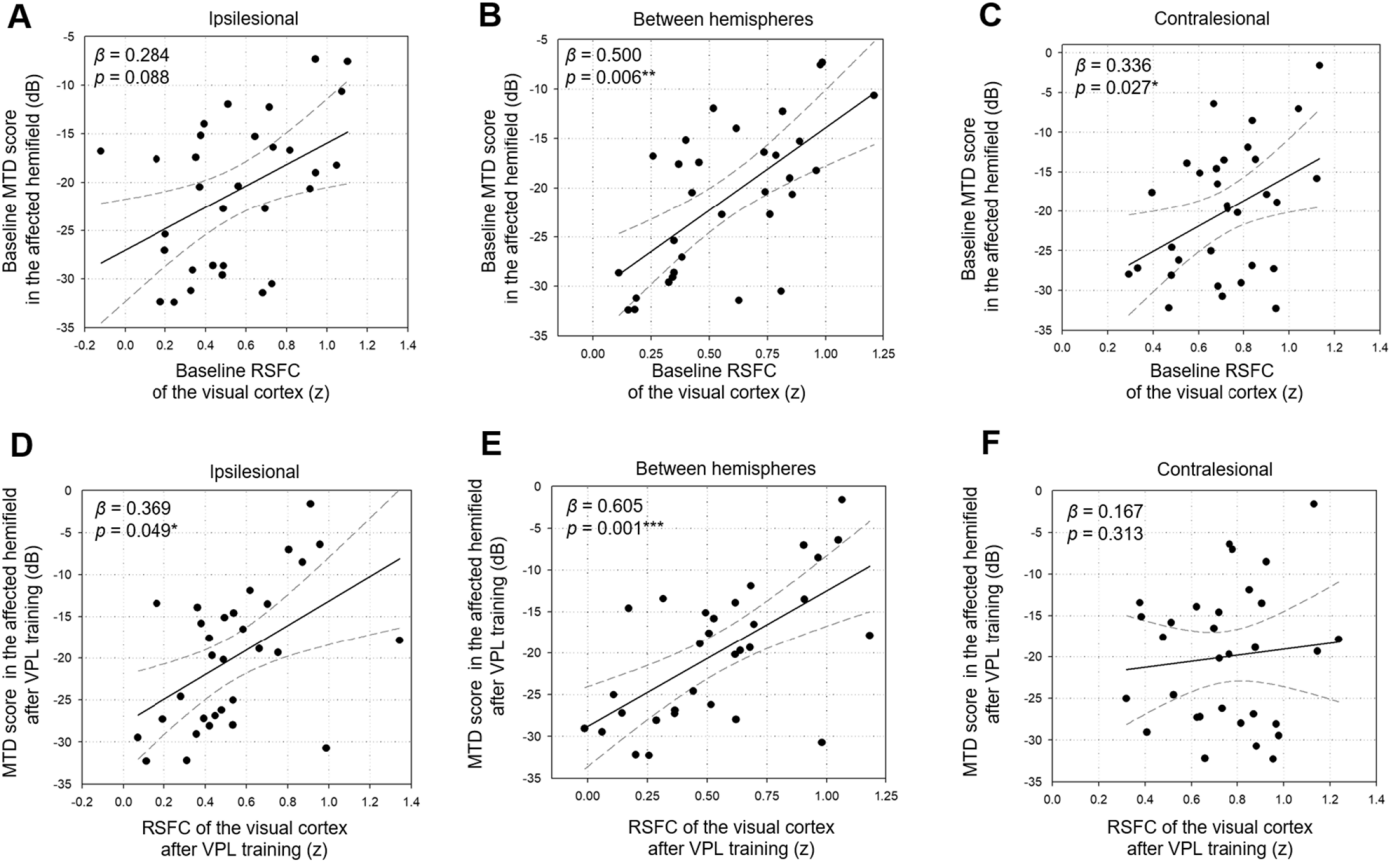
VPL-induced changes of relationships between visual RSFC and MTD score of the affected hemi-field. Pearson correlation analysis was used to assess associations between RSFCs of the visual cortex and MTD scores, and included age, sex, and lesion volume in the visual cortex as covariates. This correlation analysis was repeated for the values measured at baseline (**A**–**C**) and after the 2-month VPL training (**D**–**F**) as well as for the ipsilesional (**A**, **D**), interhemispheric (**B**, **E**), and contralesional (**C**, **F**) visual cortex. The scatter plots and the line of best fits are indicated, including visual RSFC as the x-axis and MTD score as the y-axis. The significant correlations at Bonferroni-corrected *p* < 0.0016 (0.05/3) that were corrected for three comparisons of the ipsilesional, interhemispheric, and contralesional visual cortex were indicated as the *** symbol. MTD, mean total deviation; RSFC, resting-state functional connectivity; VPL, visual perceptual learning. *p < 0.05, **p < 0.01, ***Bonferroni-corrected *p* < 0.0016.

In the training group, the interhemispheric visual RSFC was significantly associated with the mean total deviation (MTD) score at both baseline (*β* = 0.500, *p* = 0.006) and post-VPL training (*β* = 0.605, Bonferroni-corrected *p* = 0.001; Pearson correlation analysis). The association between the ipsilesional visual RSFC and the MTD score in the affected hemi-field was not significant at baseline but became significant after 2-month VPL (pre-VPL training, *β* = 0.284, *p* = 0.088; post-VPL training, *β* = 0.369, *p* = 0.049; Pearson correlation analysis). In contrast, the association between the contralesional visual RSFC and MTD scores was significant at baseline but was no longer significant after VPL (pre-VPL training, *β* = 0.336, *p* = 0.027; post-VPL training, *β* = 0.167, *p* = 0.313; Pearson correlation analysis; Fig. 2).

In the control group, the abovementioned neurobehavioral associations were not observed (Supplementary Results). The associations between the visual RSFC within the bilateral four regions-of-interests (ROIs) and MTD scores in relation to VPL are presented in Supplementary Table 1.

### Predictive brain biomarker: associations of baseline visual RSFC with the post-VPL visual performance

Whether baseline visual RSFC, as a predictive brain biomarker, is associated with post-VPL MTD score was investigated in the training group.

**Figure 3:**
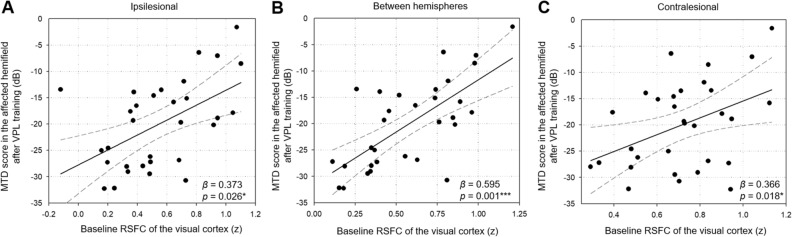
Relationships between baseline visual RSFC and post-training MTD score of the affected hemi-field. Pearson correlation analysis was used to assess associations between baseline RSFCs of the visual cortex and post-VPL MTD scores, and included age, sex, and lesion volume in the visual cortex as covariates. This correlation analysis was repeated for the ipsilesional (**A**), interhemispheric (**B**), and contralesional (**c**) visual cortex. The scatter plots and the line of best fits are indicated, including visual RSFC as the x-axis and MTD score as the y-axis. The significant correlations at Bonferroni-corrected *p* < 0.0016 (0.05/3) that were corrected for three comparisons of the ipsilesional, interhemispheric, and contralesional visual cortex were indicated as the *** symbol. MTD, mean total deviation; RSFC, resting-state functional connectivity; VPL, visual perceptual learning. *p < 0.05, **p < 0.01, ***Bonferroni-corrected *p* < 0.0016.

In the training group, the mean RSFC of the interhemispheric visual regions at baseline showed a strong positive association with the MTD score in the affected hemi-field following VPL (interhemispheric, *β* = 0.595, Bonferroni-corrected *p* = 0.001; Pearson correlation analysis; Fig. 3B). The baseline mean RSFC of the ipsilesional and contralesional visual regions was positively associated with the MTD score in the affected hemi-field after VPL (ipsilesional, *β* = 0.373, *p* = 0.026; contralesional, *β* = 0.366, *p* = 0.018; Pearson correlation analysis; Fig. 3A,C).

The abovementioned associations were not observed in the control group (Supplementary Results). Associations of the baseline visual RSFC between the ROIs and the MTD scores after VPL are presented in Supplementary Table 2.

### Subgroup analysis results: effects of baseline interhemispheric visual RSFC on the VFD recovery in relation to VPL

The two subgroups were categorized within the training group based on baseline interhemispheric visual RSFC using the K-means^++^ clustering. The MTD score at baseline and for VPL-induced changes were investigated for between- and within- subgroups to examine effects of baseline interhemispheric visual RSFC on VPL-induced VFD recovery.

**Figure 4:**
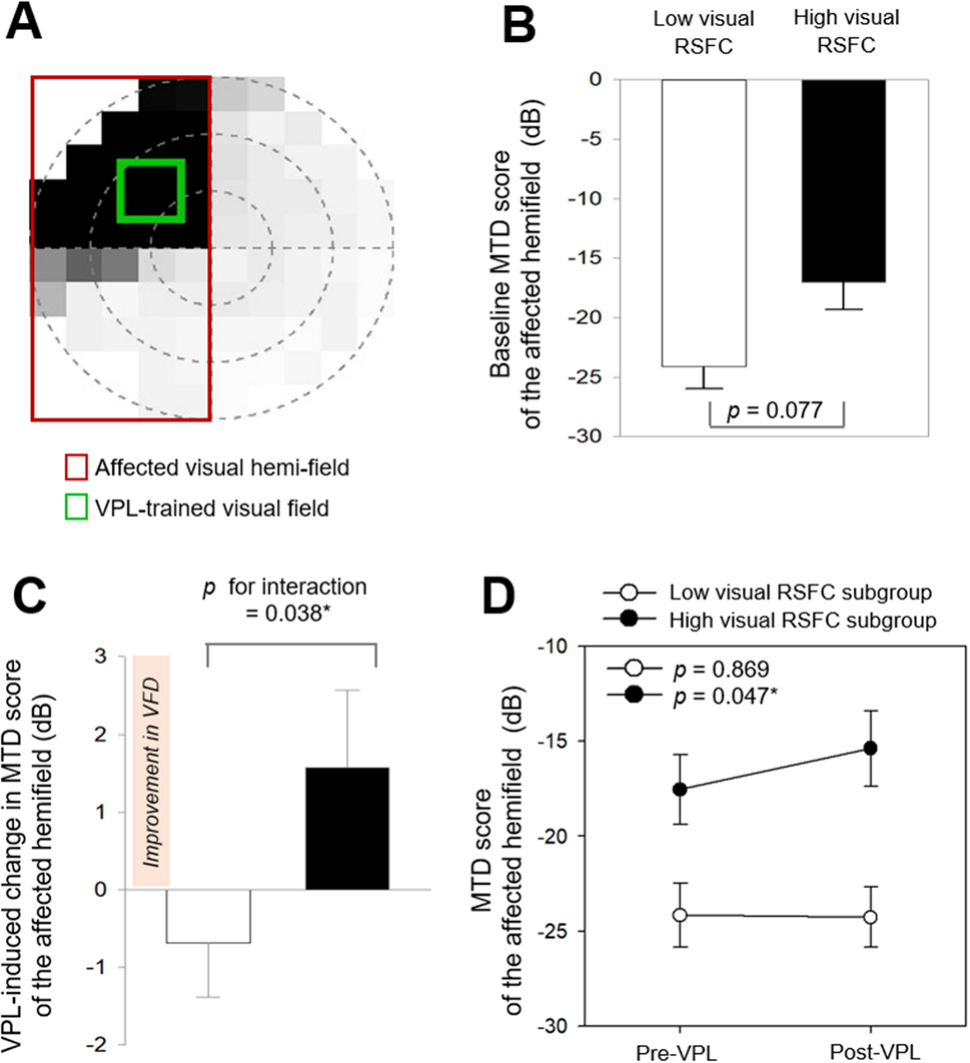
Differential VPL-induced recovery of visual field defect between subgroups based on baseline interhemispheric visual RSFC. (**A**) The affected hemi-field (red) and VPL-trained location (green) are visualized for an exemplary patient with quadrantanopia. (**B**) Baseline MTD scores were compared between subgroups based on baseline interhemispheric visual RSFC using analysis of covariance with age, sex, and lesion volume in the visual cortex as covariates. (**C**) A linear mixed-effects model for repeated measures was used to examine the fixed effects of the subgroups (high vs. low visual RSFC subgroups), time (pre-VPL vs. post-VPL), and their interaction on the MTD score. A random subject effect, age, sex, lesion volume in the visual cortex, and respective baseline values were included into the model. (**D**) The MTD score was compared at pre- and post- VPL in each of the subgroup based on baseline interhemispheric visual RSFC using paired-t tests. MTD, mean total deviation; RSFC, resting-state functional connectivity; VPL, visual perceptual learning. *p < 0.05, **p < 0.01, ***p < 0.001.

At baseline, the MTD score in the affected hemi-field showed non-significant differences between the high (n = 16) and low (n = 15) visual RSFC subgroups (*p* = 0.077; analysis of covariance), categorized using the K-means^++^ clustering based on the baseline interhemispheric visual RSFC (Fig. 4A,B). The interaction between time (pre-VPL vs. post-VPL) and the visual RSFC subgroups (high vs. low) was significant on changes in the MTD score in the affected hemi-field (*z* = 2.08, *p* for interaction = 0.038; a linear mixed-effects model for repeated measures; Fig. 4C). However, the subgroup effect according to baseline interhemispheric visual RSFC (high vs. low)(*z* = 0.64, *p* = 0.521) and the time effect (pre-VPL vs. post-VPL)(*z* = -0.14, *p* = 0.886) was not significant on changes in the MTD score in the affected hemi-field. The MTD score of the affected hemi-field improved after VPL in the high visual RSFC subgroup (*p* = 0.047; paired t-test), but not in the low visual RSFC subgroup (*p* = 0.869; Fig. 4D).

The abovementioned findings from the subgroup analysis on the MTD score were not observed in the control group (Supplementary Results).

### Subgroup analysis results: effects of baseline interhemispheric visual RSFC on the brain in relation to VPL

The visual RSFC at baseline and for VPL-induced changes were investigated for between- and within- subgroups (categorized based on baseline interhemispheric visual RSFC using the K-means^++^ clustering) to examine effects of baseline interhemispheric visual RSFC on VPL-induced RSFC changes in the visual cortex.

**Figure 5:**
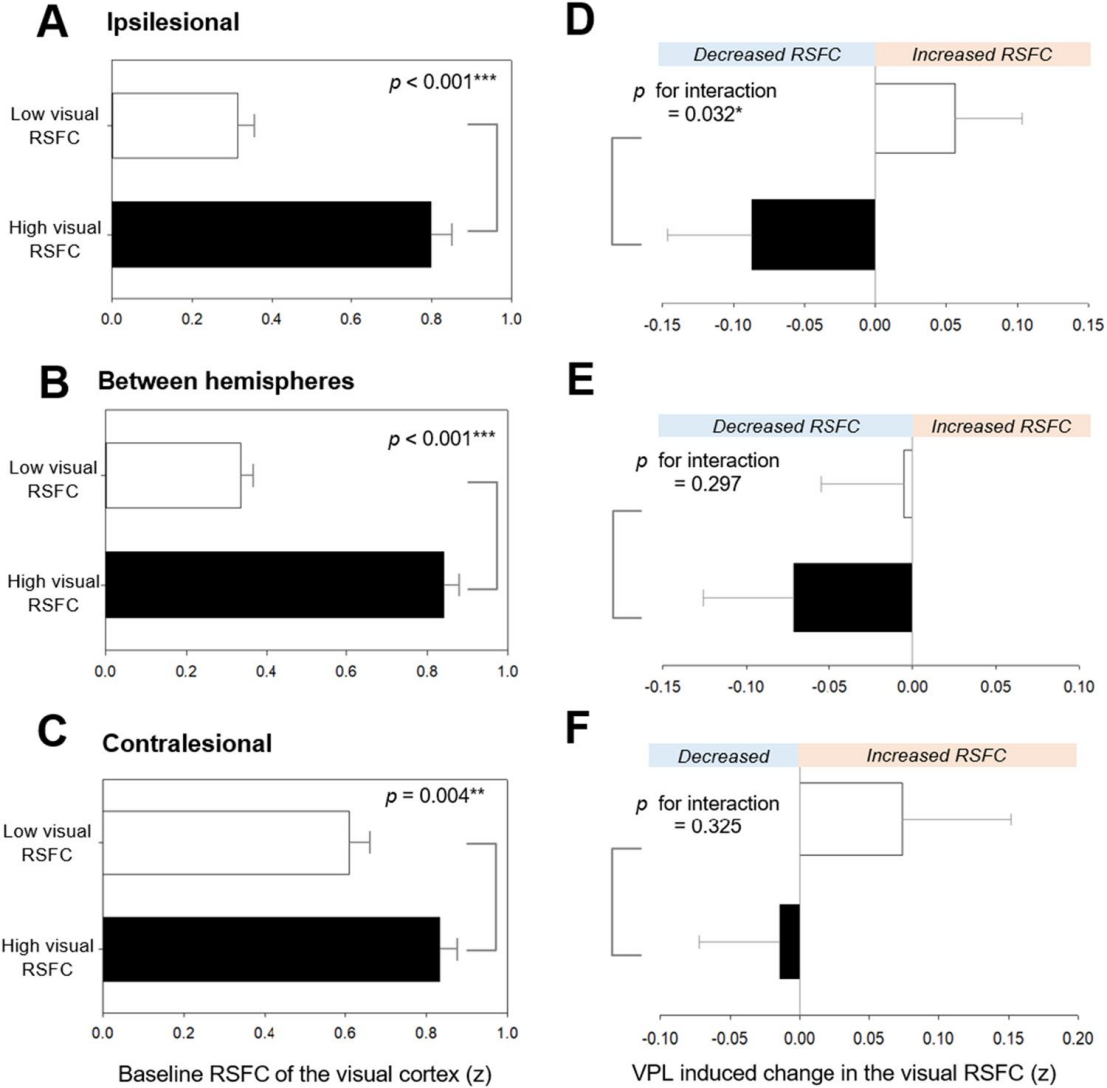
Differential VPL-induced changes in visual RSFC between subgroups based on baseline interhemispheric visual RSFC. Baseline RSFC of the visual cortex were compared between subgroups based on baseline interhemispheric visual RSFC using analysis of covariance with age, sex, and lesion volume in the visual cortex as covariates. This was repeated for (**A**) ipsilesional, (**B**) interhemispheric, (**C**) contralesional visual cortex. A linear mixed-effects model for repeated measures was used to examine the fixed effects of the subgroups (high vs. low visual RSFC subgroups), time (pre-VPL vs. post-VPL), and their interaction on RSFC of the visual cortex. A random subject effect, age, sex, lesion volume in the visual cortex, and respective baseline values were included into the model. This was repeated for (**D**) ipsilesional, (**E**) interhemispheric, (**F**) contralesional visual cortex. The significant correlations at Bonferroni-corrected *p* < 0.0016 (0.05/3) that were corrected for three comparisons of the ipsilesional, interhemispheric, and contralesional visual cortex were indicated as the *** symbol. RSFC, resting-state functional connectivity; VPL, visual perceptual learning. *p < 0.05, **p < 0.01, ***Bonferroni-corrected *p* < 0.0016.

At baseline, the mean RSFCs of the ipsilesional, interhemispheric, and contralesional visual regions were compared between the high (n = 16) and low (n = 15) visual RSFC subgroups categorized using the K-means^++^ clustering based on the baseline interhemispheric visual RSFC. Enhanced RSFC was observed in the ipsilesional (Bonferroni-corrected *p* < 0.001; analysis of covariance), interhemispheric (Bonferroni-corrected *p* < 0.001), and contralesional (*p* = 0.004) visual regions of the high visual RSFC subgroup compared to those of the low visual RSFC subgroups (Fig. 5A–C).

The interaction between time (pre-VPL vs. post-VPL) and the visual RSFC subgroups (high vs. low) was significant in the ipsilesional visual RSFC (*z* = − 2.15, *p* for interaction = 0.032; a linear mixed-effects model for repeated measures; Fig. 5D). However, the subgroup effect according to baseline interhemispheric visual RSFC (high vs. low)(*z* = 0.36, *p* = 0.721) and the time effect (pre-VPL vs. post-VPL)(*z* = 1.21, *p* = 0.227) was not significant on changes in the ipsilesional visual RSFC. In the post-hoc analyses, VPL-induced changes in the ipsilesional visual RSFC were not significant both in the high (*p* = 0.162; paired t-test) and low (*p* = 0.254) visual RSFC subgroups. The interactions between time and the visual RSFC groups were not significant in the interhemispheric (*z* = − 1.04, *p* for interaction = 0.297; a linear mixed-effects model for repeated measures; Fig. 5E) and the contralesional (*z* = − 0.980, *p* for interaction = 0.325; Fig. 5F) visual RSFC.

The abovementioned subgroup analysis results on the brain regarding visual RSFC was not observed in the control group (Supplementary Results). The baseline visual RSFC differences between the ROIs and subgroup-by-visit interactions on visual RSFC between the ROIs are shown in Supplementary Table 3.

### Neural plasticity: associations between VPL-induced changes in visual RSFC and visual performance

The associations between VPL-induced changes in the visual RSFC and those in the MTD scores were investigated as the neural plasticity underlying VPL in the training group.

**Figure 6:**
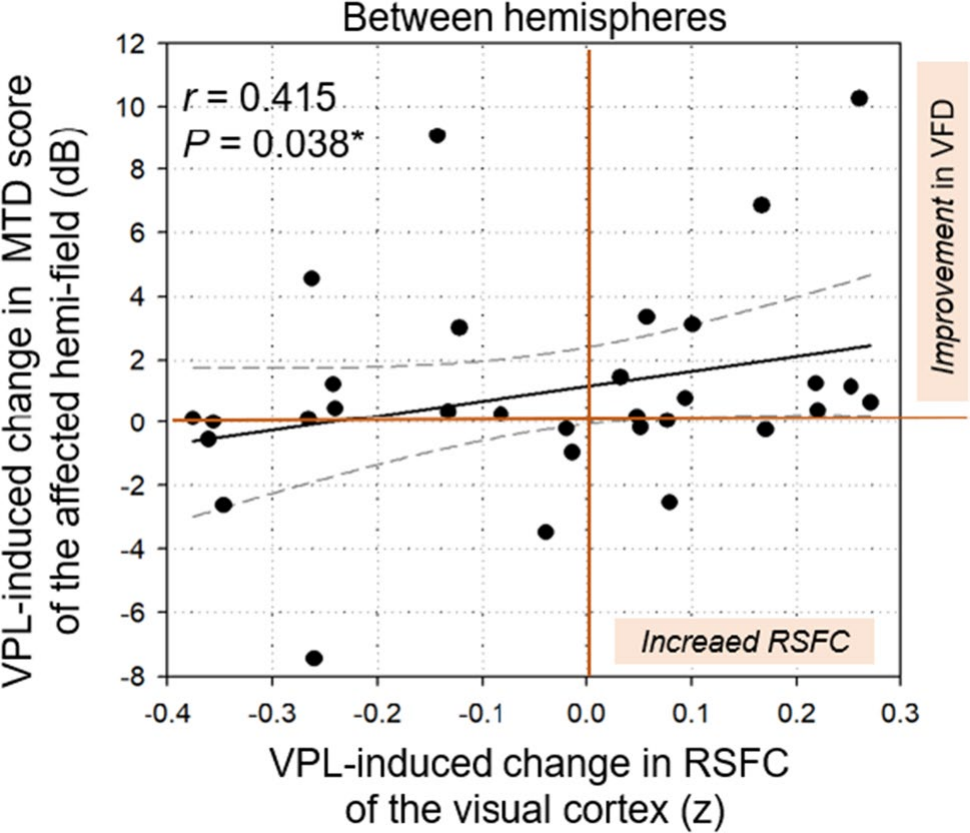
Relationships between VPL-induced changes in the interhemispheric visual RSFC and MTD score. Spearman correlation analysis was used to examine correlation between VPL-induced change in the interhemispheric visual RSFC and VPL-induced change in the MTD score of the affected hemi-field within the training group. Age, sex, and lesion volume in the visual cortex were included as covariates. MTD, mean total deviation; RSFC, resting-state functional connectivity; VPL, visual perceptual learning. *p < 0.05, **p < 0.01, ***Bonferroni-corrected *p* < 0.0016.

In the training group, VPL-induced change in the mean RSFC of the interhemispheric visual regions (*r* = 0.415, *p* = 0.038; Spearman correlation analysis) was positively associated with VPL-induced change in the MTD score of the affected hemi-field, after adjusting for age, sex, and lesion volume in the visual cortex (Fig. 6). However, VPL-induced changes in the mean RSFC of the ipsilesional (*r* = − 0.007, *p* = 0.971; Spearman correlation analysis) and contralesional (*r* = 0.180, *p* = 0.355) visual regions were not associated with VPL-induced change in the MTD score of the affected hemi-field.

We did not observe similar neuroplasticity in the control group (Supplementary Results). Associations between the VPL-induced changes in visual RSFC between the ROIs and the MTD scores are shown in Supplementary Table 4.

## Discussion

The current study suggests that higher baseline interhemispheric RSFC in the visual cortex could potentially predict greater VPL-induced VFD recovery in patients with chronic stroke patients. Notably, the baseline interhemispheric visual RSFC and VPL played an interactive role to improve VFD after VPL. Specifically, VFD was improved after VPL in the subgroup with higher baseline interhemispheric visual RSFC than in the subgroup with its lower baseline value. Moreover, higher interhemispheric visual RSFC at baseline showed the strongest association with greater MTD score after 2-month VPL training. The present findings provided critical insights regarding the neural plasticity and neurobehavioral associations underlying VPL-induced recovery of VFD in chronic ischemic stroke.

After 2-month VPL training, the positive association of the MTD score in the affected hemifield remained strongly significant with the interhemispheric visual RSFC; The positive associations of the MTD score in the affected hemifield became significant with the ipsilesional visual RSFC and became non-significant with the contralesional visual RSFC (Fig. 2). In tandem with previous literature^26–28^, a lower ipsilesional RSFC compared to contralesional visual RSFC at baseline (ipsilesional, 0.549 ± 0.306; contralesional, 0.718 ± 0.216; *p* = 0.014) was found in our study. Stroke-related dysfunction is believed to be related to weaker ipsilesional function^23,24^, accompanied by compensatory functional increase in the contralesional hemisphere. Accordingly, a compensatory increase at 1 week and a decrease at 3 months post-stroke onset were observed in the contralesional visual regions in relation to spontaneous recovery of acute stroke-related VFD^25^. Competitive interactions between the contralesional and ipsilesional hemispheres are also suggested to significantly affect stroke-related functional recovery^29,30^. The interhemispheric visual RSFC, which maintains strong neurobehavioral associations with the MTD score, may play a key role in recovering the compensatory neurobehavioral associations from the contralesional to ipsilesional visual regions. Thus, VPL may strengthen and normalize the neurobehavioral association between the ipsilesional visual RSFC and the MTD score, improving visual dysfunction. Supporting this speculation, the functional shift from the contralesional to ipsilesional was reported to underlie stroke recovery^31–34^.

**Figure 7:**
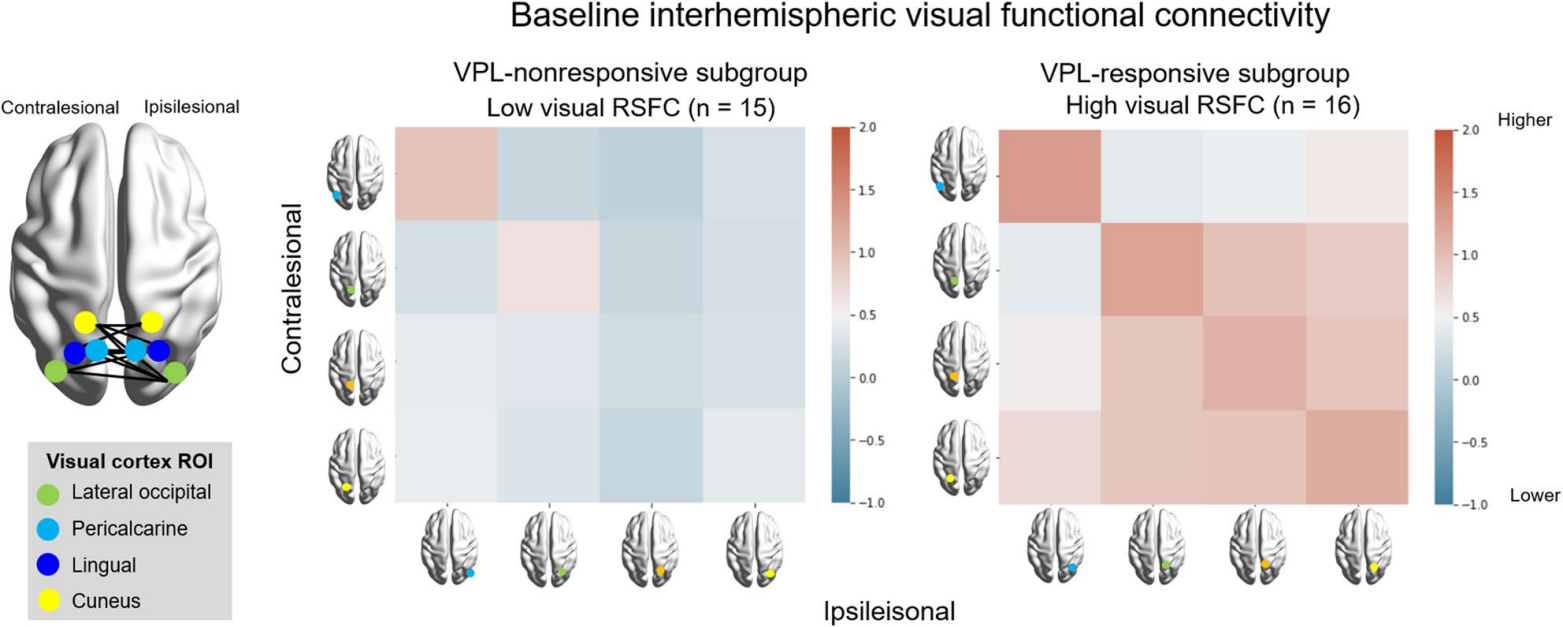
Differential VPL responsiveness in subgroup categorized based on baseline interhemispheric visual functional connectivity. K-^++^ means clustering was used for subgroup categorization according to baseline interhemispheric visual functional connectivity based on the bilateral four visual ROIs in the visual cortex (green, lateral occipital; light blue, pericalcarine; dark blue, lingual; yellow, cuneus). The four-by-four matrix indicates baseline visual functional connectivity between the contralesional (vertical) and ipsilesional (horizontal) ROIs. The red color indicates higher functional connectivity of the interhemispheric visual cortex, while the blue color indicates its lower value. High visual RSFC subgroup (n = 16) showed VFD recovery after VPL, while low visual RSFC subgroup did not (n = 15). ROI, regions-of-interests, RSFC, resting-state functional connectivity; MRI, magnetic resonance imaging; VPL, visual perceptual learning.

The current findings highlight the predictive value of baseline interhemispheric visual RSFC for VPL-induced VFD recovery (Fig. 7). Specifically, the strongest positive association between the baseline interhemispheric visual RSFC and post-VPL MTD score (Fig. 3B) showed greater VPL-induced visual improvement in the high baseline interhemispheric visual RSFC subgroup (Fig. 4C and D). The significance of these results remained unchanged after replacing the lesion volume in the visual cortex with the whole lesion volume, implicating the extent and location of the lesion (within vs. outside the occipital lobe) as non-influential factors for VPL responsiveness. The intact interhemispheric visual RSFC is a crucial player in ipsilesional visual function restoration, which is critical in improving VFD^23–25,30^. This rationale is further supported by the between-subgroup differences in the VPL-induced changes of the ipsilesional visual RSFC (Fig. 5D). That the ipsilesional visual RSFC showed decreasing tendency in the high visual RSFC subgroup (*p* = 0.162) contradicts the notion that increased ipsilesional visual RSFC underlies VFD improvement. Albeit without statistical significance; this discrepancy could be explained by two speculative hypotheses: (1) VPL may result in sharpened tuning characteristics of the visual cortical neurons^35–38^; and (2) inter-individual variations in the magnitude and direction of VPL-induced changes in visual performance and RSFC may be correlated^39–41^.

Similarly, perceptual learning of orientation discrimination tasks lowered visual functional activation and improved visual performance in previous literature^35–38^, suggesting that more specific changes may occur in fewer visual cortical neurons^35–38^. Furthermore, the VPL-responsive subgroup (high visual RSFC subgroup) in our study have efficiently restored ipsilesional dysfunction, not necessarily via increased ipsilesional visual RSFC (Fig. 5D)^42^, but after disentangling the neurobehavioral disassociations underlying VFD (Fig. 2)^26–28^. Contrastingly, the VPL-nonresponsive subgroup (low visual RSFC subgroup) may have maintained increasing contralesional RSFC (*p* = 0.358; Fig. 5F) as compensation than ipsilesional functional usage, further increasing inter-hemispheric functional imbalance^43^.

Supporting this predictive brain biomarker (Fig. 7), interactions between baseline RSFC of the interhemispheric visual cortex and VPL, rather than individually contributing, was identified to contribute VFD recovery (Fig. 4C) and RSFC changes in the ipsilesional visual cortex (Fig. 5D), adjusting for age, gender, lesion volume in the visual cortex, and respective baseline MTD or RSFC. Accordingly, in our auxiliary analysis, baseline visual RSFCs were significantly correlated with post-VPL MTD scores, but not with VPL-induced changes in MTD scores. These results indicate that VFD recovery cannot be solely accounted by VPL alone, but to interrelationships between high baseline RSFC of the interhemispheric visual cortex and VPL. Consistent with the previous literature^44^, VPL-induced change in the MTD scores was not dependent upon patient age, time since stroke, baseline MTD score, baseline visual RSFCs, and lesion volume (visual cortex and whole brain), despite the baseline interhemispheric visual RSFC being the strongest factor (*p* < 0.20 at backward stepwise multiple regression analysis). Our supplementary subgroup analyses further support that baseline MTD and lesion volume, in interactions with VPL, may not predict VPL-induced VFD recovery. Yet considering baseline negative association of lesion volume with interhemispheric visual RSFC and baseline MTD score (Supplementary Results), prompt individualized rehabilitation strategies for VFD should be developed based on the interhemispheric visual RSFC, while considering the volume of stroke ischemic lesion or VFD severity, at baseline.

The study showed that VPL-induced increases in the interhemispheric visual RSFC are positively associated with MTD scores of the affected hemi-field, suggesting that neural plasticity underlies VFD recovery (Fig. 6). This correlation remained significant with additional covariates of baseline MTD and whole brain lesion volume. This is consistent with the residual visual activation theory proposed by vision-restoration studies^45^: experience-dependent training such as VPL strengthens synaptic synchronization of partially lesioned brain regions and reorganizes the brain network through axonal pruning and sprouting of the pyramidal cells^24,42,46^. VPL, which requires visual discrimination and decision making for promptly recognizing previously seen letters and orientations^5–8^, may upregulate synapse formation as well as neural proliferation within the interhemispheric visual cortex, as manifested by learning-dependent increases in blood-oxygen-level-dependent signals^42,47,48^. However, further studies investigating underlying cellular and molecular mechanisms are needed to clarify whether neuronal regeneration or vascular supply underlies VPL-induced increases in the interhemispheric visual RSFC^42,49,50^. Despite inter-individual variations, increased interhemispheric visual RSFC underlying VPL-induced VFD recovery^42,48^ may play a pivotal role in restoring visuospatial function through regulating functional balance between the ipsilesional and contralesional visual regions^23–25^.

The following limitations should be considered when interpreting the present study’s findings. To explore intrinsic functional brain changes regarding the lesion in the visual cortex, RSFC changes within the visual cortex were emphasized in relation to VPL-induced VFD recovery in stroke. As the posterior cerebral artery supplies the extensive regions including the splenium of corpus callosum and medial temporal lobe in addition to the occipital lobe, the interactive role between the brain regions at the whole-brain level with relation to VPL-induced VFD recovery warrants further investigation in posterior cerebral artery stroke^51,52^. Moreover, future multi-modal neuroimaging studies, which use resting-state fMRI in addition to the diffusion tensor imaging measuring structural connectivity and the perfusion-weighted imaging assessing cerebral blood flow, may provide more holistic viewpoint on the neurobiological mechanisms underlying VPL-induced recovery of VFD in stroke. Although eye tracking could not be used in the study, we excluded subjects with more than 20% fixation losses in the Humphrey Visual Field (HVF) tests from the analysis. Another limitation is the lack of follow-up on the RSFC and MTD scores after 2-month VPL training completion. Future longitudinal investigations using eye-tracking may provide further insights into the long-term trajectories of the visual RSFC and VFD recovery in relation to VPL for chronic ischemic stroke.

The present study highlights critical insights regarding the neural plasticity, neurobehavioral associations, and predictive brain biomarker of VPL-induced recovery of VFD in chronic ischemic stroke. Importantly, the baseline interhemispheric visual RSFC and VPL played an interactive role to improve VFD after VPL: high interhemispheric visual RSFC may allow recovery of compensatory neurobehavioral associations from the contralesional to ipsilesional visual regions, thereby leading to VPL-induced visual improvement. Thus, increasing RSFC of the interhemispheric visual cortex could be a potentially promising target for future prevention and treatment strategies of stroke-related VFD, including neuromodulation and VPL-based rehabilitation therapies such as virtual reality training. Further, longer sessions and duration of VPL can also be recommended to patients with stroke-related VFD with lower interhemispheric visual RSFC at baseline, while considering lesion volume and VFD severity. In conclusion, it is crucial to develop early individualized rehabilitation plans that consider the baseline interhemispheric visual RSFC as a predictive biomarker for training responsiveness.

## Methods

### Participants and study design

This retrospective study of our two previous studies (one published^13^ and one unpublished) analyzed patients with VFD following chronic ischemic stroke (Fig. 1A, Supplementary Fig. 1). The inclusion criteria were: (1) age > 20 years, (2) having at least 6 months of VFD symptoms, (3) a neurologist-diagnosed chronic ischemic stroke in a unilateral posterior cerebral arterial territory (confirmed by structural MRI; Supplementary Fig. 2), (4) having homonymous hemianopia or quadrantanopia, and (5) having reliable resting-state fMRI and HVF tests. The exclusion criteria were: (1) low reliability in the HVF test (more than 20% fixation losses), (2) cognitive impairments (less than 24 points of the Mini-Mental State Examination), or (3) physician-diagnosed ophthalmologic disorders.

The study design included in the final analysis is illustrated in Fig. 1A. The participants were divided into the VPL training group (n = 31) and the control group (n = 15). In the training group, 20 patients received VPL immediately after baseline evaluation, and another 11 patients received VPL training after the control period of 2 months. The final analysis included 31 patients who had 24 sessions of VPL training for 2 months and 15 patients who participated in the 2-month control period. Resting-state fMRI and HVF tests were performed at baseline and 2-month follow-up.

The patients who only participated in the VPL (n = 20) were older than those in the control group (n = 15) (*p* = 0.020); however, the two groups showed no differences in other characteristics including the proportion of sex (*p* = 0.265), hemisphere with infarct (*p* = 0.313), and pattern of the visual field defect (*p* = 0.433), as well as time from stroke onset (*p* = 0.076), the whole stroke lesion volume (*p* = 0.158), and the lesion volume in the visual cortex only (*p* = 0.117). Written informed consent was provided by all participants or their legal representatives and the study protocol was approved by the Institutional Review Board of Asan Medical Center. The study was conducted in accordance with the Declaration of Helsinki.

### Visual perceptual learning and humphrey visual field test

The VPL training was elicited using an orientation discrimination test with horizontal-vertical grating (Fig. 1B). The visual stimuli were presented on the 37 cm wide screen, which was 40 cm away from the participant with chinrest. After 500 ms of the ready screen (empty circle on the fixation location), the target screen was presented for 150 ms duration. Based on the baseline HVF tests, single location training of either upper or lower quadrant was provided for patients with quadrantanopia (n = 18) and two location training of both upper and lower quadrants was provided for patients with hemianopia (n = 13). Each training session (26 trials for each defective quadrant and 6 trials for each normal quadrant for each run; patients performed 6 runs in each training session) consisted of 264 trials trials (6 × [26 × 1 + 6 × 3] for 1 defective and 3 normal quadrants) for patients with quadrantanopia and 384 trials (6 ×  [26 × 2 + 6 × 2] for 2 defective and 2 normal quadrants) for patients with hemianopia.

The target screen consisted of a centrally located fixation letter ("ㅋ" or "ㅌ" in Korean language) and a peripherally positioned texture square (horizontal or vertical grating pattern). The orientation stimuli were randomly presented in a random location for the normal quadrant or a fixed location for the individualized defective quadrant of the visual field based on baseline HVF test results. Within the quadrants, the peripherally positioned texture squares were primarily centered 10 degrees away from the fixation point at the center of the display, but they were adjusted towards the peripheral side if the VFD predominantly affected the edge of the affected hemifield, as determined by the baseline HVF tests. Its size was 10º x 10º, and it was filled with either grating patterns of 2.5 Hz frequency of either horizontally or vertically oriented sine waves.

During each response screen, the patients were asked to identify the fixation letter on the center (“ᄏ” or “ᄐ” in Korean) and the direction of the orientation of grating (horizontal or vertical) of the texture square by pressing two of the four response buttons, while fixating their eyes on the center of the stimuli display. The patients were presented with < 8000 ms of the response screen (< 2000 ms for the fixation letter decision and < 6000 ms for the orientation decision), immediately followed by the next ready screen.

Visual field defect was quantitatively assessed using a Humphrey field analyzer (HFA 750i, Zeiss-Humphrey, Leandro, CA, USA), which employed the light detection task and central 30–2 threshold SITA-Fast protocol to assess the visual fields of each eye. The patients were asked to immediately respond when the short light stimuli appeared in their line of sight. The total deviation scores, indicating light detection performance at each tested retinal point, in reference to age-matched normative values, were provided as an output of the HFA. The MTD score was calculated by averaging the total deviation scores in the affected hemi-field, averaged across eyes (e.g., the affected hemi-field is the left for left quadrantanopia or hemianopia patients).

### Analysis of resting-state fMRI data

For resting-state fMRI data analysis, 115 volumes were used after removing the first five volumes to achieve signal equilibrium. The pre-processing of resting-state fMRI images was performed using the CONN toolbox (https://web.conn-toolbox.org)^53^ and Statistical Parametric Mapping 12 (SPM12; Wellcome Department of Cognitive Neurology, Institute of Neurology, London, UK; http://www.fil.ion.ucl.ac.uk/spm). The pre-processing steps are described in Supplementary Methods for detail.

Among 42 parcellated gray matter regions based on FreeSurfer^54^, the following four ROIs in the occipital lobe were selected given its critical role in visual perception: cuneus cortex; pericalcarine cortex; lingual gyrus; and lateral occipital cortex (Fig. 7; Supplementary Fig. 2). After averaging the time series of all voxels within each ROIs, the bivariate correlation coefficients between each pair of ROIs were calculated and converted to z-scores using Fisher’s transformation. The mean RSFCs of the ipsilesional, interhemispheric, and contralesional visual regions were computed by averaging a total of 6 ipsilesional, 16 interhemispheric, and 6 contralesional pairs of RSFC, respectively.

### Subgroup analysis based on the baseline interhemispheric RSFC

The K-means^++^ clustering based on the baseline interhemispheric RSFCs in the occipital lobe was performed for subgroup analysis in both the VPL training and control groups^55^. The silhouette coefficients of K-means^++^ clustering was 0.661 and 0.604 for the training and control groups, respectively, indicating satisfactory clustering of the subgroups^55^. In the training group, the 31 patients with VFD were categorized into the high visual RSFC subgroup (n = 16) and low visual RSFC subgroup (n = 15). In the control group, the 15 patients with VFDs were categorized into the high (n = 6) and low (n = 9) visual RSFC subgroups. The high visual RSFC subgroups exhibited larger stroke lesion volumes in the visual cortex and whole brain, in both the training and control groups. However, there were no significant differences between the subgroups in terms of other characteristics (Supplementary Table 5).

### Statistical analyses

Normality of the data was assessed using the Shapiro–Wilks test. Continuous and categorical demographic and clinical characteristics were compared between the two groups (VPL training vs. control groups; high vs. low visual RSFC subgroups within the training group; high vs. low visual RSFC subgroups within the control group) using an independent t-test or chi-squared test.

Within the VPL and control groups, Pearson or Spearman correlation analysis was used to examine associations between the visual RSFC and the MTD score in the affected hemi-field, at baseline and 2-month follow-up, and for changes during the 2-month period. Age, sex, and lesion volume in the visual cortex were included as covariates.

For all the VPL and control subgroups, analysis of covariance models, with age, sex, and lesion volume in the visual cortex as covariates, were used to assess between-group differences in the visual RSFC and the MTD score at baseline. To investigate changes during 2-month period according to the baseline interhemispheric visual RSFC, a linear mixed-effects model for repeated measures was used to examine the fixed effects of the subgroups (high vs. low visual RSFC subgroups), time (baseline vs. 2 month-follow-up), and their interaction with the visual RSFC and the MTD score. A random subject effect, age, sex, lesion volume in the visual cortex, and respective baseline values were included also into the model. Post-hoc paired t-tests were performed within the subgroups.

All the statistical tests were two-tailed, with a statistical threshold of *p* < 0.05. All statistical analysis was performed using the Stata SE (Stata Corp, College Station, TX, USA). The Bonferroni-corrected threshold of *p* < 0.0016 (0.05/3) was also used for three comparisons of the ipsilesional, interhemispheric, and contralesional visual cortex.

### Supplementary Information

Supplementary Information.

## Data Availability

The deidentified data can be shared upon reasonable request and a methodologically sound proposal upon the corresponding author.

## References

[1] Pollock A (2011). Interventions for visual field defects in patients with stroke. Cochrane Database Syst. Rev..

[2] Pambakian A, Kennard C (1997). Can visual function be restored in patients with homonymous hemianopia?. Br. J. Ophthalmol..

[3] Zhang X, Kedar S, Lynn M, Newman N, Biousse V (2006). Homonymous hemianopias: Clinical–anatomic correlations in 904 cases. Neurology.

[4] Zhang X, Kedar S, Lynn M, Newman N, Biousse V (2006). Natural history of homonymous hemianopia. Neurology.

[5] Beste C, Dinse HR (2013). Learning without training. Curr. Biol..

[6] Karni A, Sagi D (1993). The time course of learning a visual skill. Nature.

[7] Sasaki Y, Nanez JE, Watanabe T (2010). Advances in visual perceptual learning and plasticity. Nat. Rev. Neurosci..

[8] Yotsumoto Y, Watanabe T, Sasaki Y (2008). Different dynamics of performance and brain activation in the time course of perceptual learning. Neuron.

[9] Huxlin KR (2009). Perceptual relearning of complex visual motion after V1 damage in humans. J. Neurosci..

[10] Saionz EL, Tadin D, Melnick MD, Huxlin KR (2020). Functional preservation and enhanced capacity for visual restoration in subacute occipital stroke. Brain.

[11] Cavanaugh MR, Barbot A, Carrasco M, Huxlin KR (2019). Feature-based attention potentiates recovery of fine direction discrimination in cortically blind patients. Neuropsychologia.

[12] Das A, Tadin D, Huxlin KR (2014). Beyond blindsight: Properties of visual relearning in cortically blind fields. J. Neurosci..

[13] Lee EJ (2023). Digital therapeutics with visual discrimination training for cortical blindness in patients with chronic stroke. J. Stroke.

[14] Pollock A (2019). Interventions for visual field defects in people with stroke. Cochrane Database Syst. Rev..

[15] Raninen A, Vanni S, Hyvärinen L, Näsänen R (2007). Temporal sensitivity in a hemianopic visual field can be improved by long-term training using flicker stimulation. J. Neurol. Neurosurg. Psychiatry.

[16] Saionz EL, Busza A, Huxlin KR (2022). Rehabilitation of visual perception in cortical blindness. Handb. Clin. Neurol..

[17] Furmanski CS, Schluppeck D, Engel SA (2004). Learning strengthens the response of primary visual cortex to simple patterns. Curr. Biol..

[18] Schwartz S, Maquet P, Frith C (2002). Neural correlates of perceptual learning: A functional MRI study of visual texture discrimination. Proc. Natl. Acad. Sci. U. S. A..

[19] Walker MP, Stickgold R, Jolesz FA, Yoo S-S (2005). The functional anatomy of sleep-dependent visual skill learning. Cereb. Cortex.

[20] Lewis CM, Baldassarre A, Committeri G, Romani GL, Corbetta M (2009). Learning sculpts the spontaneous activity of the resting human brain. Proc. Natl. Acad. Sci. U S A..

[21] Henriksson L, Raninen A, Näsänen R, Hyvärinen L, Vanni S (2007). Training-induced cortical representation of a hemianopic hemifield. J. Neurol. Neurosurg. Psychiatry.

[22] Baldassarre A (2012). Individual variability in functional connectivity predicts performance of a perceptual task. Proc. Natl. Acad. Sci. U. S. A.

[23] Carter AR (2010). Resting interhemispheric functional magnetic resonance imaging connectivity predicts performance after stroke. Ann. Neurol..

[24] Dilks DD, Serences JT, Rosenau BJ, Yantis S, McCloskey M (2007). Human adult cortical reorganization and consequent visual distortion. J. Neurosci..

[25] Kim YH (2019). Early functional connectivity predicts recovery from visual field defects after stroke. J. Stroke.

[26] Goebel R, Muckli L, Zanella FE, Singer W, Stoerig P (2001). Sustained extrastriate cortical activation without visual awareness revealed by fMRI studies of hemianopic patients. Vision Res..

[27] Nelles G (2002). Brain representation of hemifield stimulation in poststroke visual field defects. Stroke.

[28] Schoenfeld MA (2002). Analysis of pathways mediating preserved vision after striate cortex lesions. Ann. Neurol..

[29] Heiss WD, Kessler J, Thiel A, Ghaemi M, Karbe H (1999). Differential capacity of left and right hemispheric areas for compensation of poststroke aphasia. Ann. Neurol..

[30] Murase N, Duque J, Mazzocchio R, Cohen LG (2004). Influence of interhemispheric interactions on motor function in chronic stroke. Ann. Neurol..

[31] Kim YH (2006). Longitudinal fMRI study for locomotor recovery in patients with stroke. Neurology.

[32] Puh U, Vovk A, Sevšek F, Šuput D (2007). Increased cognitive load during simple and complex motor tasks in acute stage after stroke. Int. J. Psychophysiol.

[33] Tombari D (2004). A longitudinal fMRI study: In recovering and then in clinically stable sub-cortical stroke patients. Neuroimage.

[34] Ward NS (2004). Functional reorganization of the cerebral motor system after stroke. Curr. Opin. Neurol..

[35] Crist RE, Li W, Gilbert CD (2001). Learning to see: Experience and attention in primary visual cortex. Nat. Neurosci..

[36] Schiltz C (1999). Neuronal mechanisms of perceptual learning: Changes in human brain activity with training in orientation discrimination. Neuroimage.

[37] Schoups A, Vogels R, Qian N, Orban G (2001). Practising orientation identification improves orientation coding in V1 neurons. Nature.

[38] Teich AF, Qian N (2003). Learning and adaptation in a recurrent model of V1 orientation selectivity. J. Neurophysiol..

[39] Dale G, Cochrane A, Green CS (2021). Individual difference predictors of learning and generalization in perceptual learning. Atten. Percept. Psychophys.

[40] Freyer F, Becker R, Dinse HR, Ritter P (2013). State-dependent perceptual learning. J. Neurosci..

[41] Kim Y-H (2015). Real-time strategy video game experience and visual perceptual learning. J. Neurosci..

[42] Gilbert CD, Li W (2012). Adult visual cortical plasticity. Neuron.

[43] Woods M, Williamson JB, White KD, Maitland CG, Heilman KM (2017). Shifting spatial neglect with repeated line bisections: Possible role of lateralized attentional fatigue. Cogn. Behav. Neurol..

[44] Cavanaugh MR, Huxlin KR (2017). Visual discrimination training improves Humphrey perimetry in chronic cortically induced blindness. Neurology.

[45] Sabel BA, Henrich-Noack P, Fedorov A, Gall C (2011). Vision restoration after brain and retina damage: The “residual vision activation theory”. Prog. Brain Res..

[46] Baker CI, Peli E, Knouf N, Kanwisher NG (2005). Reorganization of visual processing in macular degeneration. J. Neurosci..

[47] Szwed M (2011). Specialization for written words over objects in the visual cortex. Neuroimage.

[48] Vaina LM, Belliveau JW, Des Roziers EB, Zeffiro TA (1998). Neural systems underlying learning and representation of global motion. Proc. Natl. Acad. Sci. U. S. A..

[49] Siegel JS, Shulman GL, Corbetta M (2017). Measuring functional connectivity in stroke: Approaches and considerations. J. Cereb. Blood Flow Metab..

[50] Siegel JS, Snyder AZ, Ramsey L, Shulman GL, Corbetta M (2016). The effects of hemodynamic lag on functional connectivity and behavior after stroke. J. Cereb. Blood Flow Metab..

[51] Ryu JC, Kim JS (2022). Mechanisms of stroke in patients with fetal posterior cerebral artery. J. Stroke Cerebrovasc. Dis..

[52] Lunven M (2015). White matter lesional predictors of chronic visual neglect: A longitudinal study. Brain.

[53] Whitfield-Gabrieli S, Nieto-Castanon A (2012). Conn: A functional connectivity toolbox for correlated and anticorrelated brain networks. Brain Connect..

[54] Desikan RS (2006). An automated labeling system for subdividing the human cerebral cortex on MRI scans into gyral based regions of interest. Neuroimage.

[55] Arthur D, Vassilvitskii S (2006). k-means++: The Advantages of Careful Seeding.

