# Supplementary material for: Functional connectivity interacts with visual perceptual learning for visual field recovery in chronic stroke
Source: Sci Rep. 2024 Feb 8;14:3247. doi: 10.1038/s41598-024-52778-x (PMC10853510; doi:10.1038/s41598-024-52778-x)
Supplement: Supplementary file 1 — Supplementary Information. [file 41598_2024_52778_MOESM1_ESM.docx]

**Supplementary Materials**

Functional connectivity interacts with visual perceptual learning for visual field recovery in chronic stroke

**Supplementary Methods**

**Supplementary Results**

**Supplementary Figure 1 - 2**

**Supplementary Tables 1 - 5**

**Supplementary Methods**

***Contrast level test for difficulty adjustment of VPL***

The contrast sensitivity was adjusted to reach about 70% correction ratio using the contrast level test performed after 6 or 12 visual perceptual learning (VPL) training sessions. The determined contrast levels were used for the next 6 or 12 training sessions. The task paradigm was identical to the training session, but the contrast level of the grating pattern was changed during the test, consisting of 72 trials (30 trials each for the two defective quadrants and 6 trials each for the two normal quadrants). In vision sciences, contrast level measurements are reported in a logarithmic scale, as sensory systems respond logarithmically to stimuli changes. This conversion facilitates linear scale representation for convenient statistical analysis. The contrast level (displayed contrast-to-background intensity range) for the grating pattern is indexed on a scale of two (0 to 7), with higher values indicating greater contrast discrimination sensitivity. The initial contrast level was 1 (i.e., 2^-1^, contrast-to-background intensity change=50%), and the contrast level for next trial was exponentially adjusted based on the 3-up-1-down staircase rule (changed by integer units). For instance, 3 consecutive correct responses from 50% led to a 25% contrast level, while an incorrect response from 50% led to a 100% contrast level. A contrast level of 100% (2^0^) represented the maximum contrast-to-background intensity of the gradient pattern (0–255 sine wave intensity range), while about 0.78% (2^-7^) represented the minimum contrast-to-background intensity of the gradient pattern (126–129 sine wave intensity range).

***Extraction and preprocessing of ischemic lesion***

The individual volume of the ischemic lesion was extracted for the whole brain and visual cortex based on brain magnetic resonance imaging (MRI) as follows. In our first previous study (Supplementary Fig. 1A)^1^, the ischemic lesion was extracted in the native diffusion-weighted images (DWI) space using the FSL toolbox in FMRIB^2^ and manually delineated by an investigator (Y.H.K.) under the supervision of a stroke neurologist (D.W.K.) who was blinded to the clinical information. The lesion masks in the native DWI space were co-registered to the high-resolution T1-weighted images and then normalized to the standard space. Using the inverse matrix of the abovementioned two-way registration, the Automated Anatomical Labeling template in the standard space was inversely registered to the individual T1 space, and the lesion volume was calculated in the native T1 space for each individual. In our second previous study (Supplementary Fig. 1B), the ischemic lesion was automatically segmented in the T1-weighted images using the LINDA toolbox ^3^. The lesion masks in the T1 space were normalized to the standard space, and then inversely registered into the individual T1 space using the inverse matrix for the calculation of the individual stroke ischemic volume.

Among the patients with visual field defect (VFD), the infarct location was matched to the left hemisphere by inverting the MRI scans along the x-axis for those who had infarction in the right hemisphere (VPL training group, 19 of 31 patients; control group, 8 of 15 patients). Thus, in the present study, the left and right hemisphere were considered as the ipsilesional and contralesional hemisphere, respectively (Supplementary Fig. 2A).

***Acquisition and preprocessing of brain magnetic resonance imaging data***

Resting-state functional magnetic resonance imaging (rsfMRI) data and high-resolution T1- weighted images were acquired using a Philips Achieva 3T scanner (Philips Medical Systems, Amsterdam, the Netherlands). rsfMRI data were acquired using a gradient-echo echo-planar imaging pulse sequence with the following parameters: repetition time (TR) 2,000 ms; echo time (TE) 30 ms; flip angle (FA) 90°; field-of-view (FOV) 200 mm^2^; voxel size 1.6×1.6×3.5 mm^3^; 120 volumes; and 31 slices without gap. The patients were instructed to keep their eyes closed and avoid specific thoughts during the rsfMRI scan. For co-registration with the rsfMRI scan, high-resolution T1- weighted structural images were acquired with the magnetization-prepared rapid acquisition with gradient echo sequence using the following parameters: TR 9.9 ms; TE 4.6 ms; FA 8°; and voxel size 1 × 1 × 1 mm^3^.

As the first pre-processing step, rsfMRI data were estimated and corrected for subject-level motion. The images were then smoothed using an 8-mm full-width-at-half-maximum Gaussian kernel. The resultant images were examined for outlier scans and denoised using the Artifact Detection Tools (http://www.nitrc.org/projects/artifact_detect) as follows. The head motion outlier volumes and parameters were regressed out using the criteria of: > 0.9 mm of the combination of translational and rotational voxel displacements from the previous volume; or > 5 standard deviations of the average intensity from the mean intensity of the session. To eliminate potential physiological sources of noise from white matter and cerebrospinal fluid, the anatomical component-based noise correction method was used^4^. Finally, temporal band pass filtering (0.008–0.09 Hz) was performed to remove other spurious sources of noise.

T1-weighted images were skull-stripped and parcellated into 82 gray matter regions (42 per each hemisphere) using the FreeSurfer^5^. The raw T1-weighted images were also segmented into gray matter, white matter and cerebrospinal fluid using a probabilistic tissue segmentation implemented in the FMRIB’s Automated Segmentation Tool. The gray matter, white matter, and cerebrospinal fluid images, as well as the parcellated 82 gray matter regions were linearly registered into the rsfMRI images using the inverse matrix of registration of the rsfMRI images into the T1-weighted images. These co-registered images were used for rsfMRI data processing.

***Statistical analyses***

Post-hoc analyses were conducted by repeating the statistical analyses with mean total deviation (MTD) score using resting-state functional connectivity (RSFC) of the 6 ipsilesional, 16 interhemispheric, and 6 contralesional pairs of the regions-of-interests (ROIs): Cuneus cortex; Pericalcarine cortex; Lingual gyrus; Lateral occipital cortex (Supplementary Tables 1–4; Supplementary Fig. 2C). Bonferroni correction was applied to correct for multiple comparisons (i.e., ipsilesional and contralesional pairs of RSFC, *p* < 0.05 / 6, number of comparisons = 6; interhemispheric pair of RSFC, *p* < 0.05 / 16, number of comparisons = 16).

Auxiliary analyses were conducted by investigating the associations between the participant characteristics (patient age, time since stroke, lesion volume), the MTD score in the affected hemi-field, and the mean RSFC of the ipsilesional, interhemispheric, and contralesional visual regions at baseline using Spearman correlation analyses.

The multivariate stepwise regression analysis with backward selection were performed within the VPL training group (n = 31) and control group (n = 15). The dependent variable included 2-month changes in the MTD scores of the affected hemifield. The independent variables included patient age, time since stroke, whole brain lesion volume, lesion volume in the visual cortex, baseline mean RSFC of the ipsilesional, interhemispheric, and contralesional visual regions, as well as baseline MTD in the affected hemifield.

Auxiliary subgroup analyses were performed to investigate effects of baseline MTD and lesion volume in VPL-induced VFD recovery. In the VPL training group, K-means^++^ clustering was performed based on the baseline MTD scores in the affected hemifield, lesion volume in the visual cortex, and whole brain lesion volume, respectively. The baseline MTD scores were compared between the two subgroups categorized on the baseline MTD scores, using analysis of covariance models, with age, sex, and lesion volume in the visual cortex as covariates. The baseline MTD scores were compared between the two subgroups categorized on the lesion volume in the visual cortex and whole brain lesion volume, using analysis of covariance models, with age and sex as covariates. A linear mixed-effects model for repeated measures was used to examine the fixed effects of the subgroups (high vs. low subgroups), time (baseline vs. 2 month-follow-up), and their interaction with the MTD score. A random subject effect, age, sex, and lesion volume in the visual cortex were included also into the model for the subgroup based on baseline MTD scores. A random subject effect, age, sex, and baseline MTD score were included also into the model for the subgroup based on the lesion volume in the visual cortex and whole brain lesion volume. Post-hoc paired t-tests were performed within the subgroups.

As sensitivity analyses to investigate the effects of whole brain lesion volume, Pearson or Spearman correlation analysis with age, sex, and whole brain lesion volume as covariates was used to investigate associations between the visual RSFC and the MTD score at baseline and 2-month follow-up, and for 2-month changes. A linear mixed-effects model for repeated measures was used to examine the fixed effects of the subgroups (high vs. low subgroups according to baseline interhemispheric visual RSFC), time (pre-VPL vs. post-VPL), and their interaction with the MTD score. A random subject effect, age, sex, whole brain lesion volume, and baseline MTD score were included also into the model for the subgroup based on baseline MTD scores.

As sensitivity analyses to investigate the effects of baseline MTD, Pearson or Spearman correlation analysis with age, sex, lesion volume in the visual cortex, and baseline MTD scores as covariates was used to investigate associations between the visual RSFC and the MTD score at baseline and 2-month follow-up, and for 2-month changes.

Predictive brain biomarker and neural plasticity analyses were repeated for the MTD score in the trained location. Within the VPL group, Pearson or Spearman correlation analysis was used to examine associations between the visual RSFC and the MTD score in the trained location, at baseline and 2-month follow-up, and for changes during the 2-month period. Age, sex, and lesion volume in the visual cortex were included as covariates. The baseline MTD scores in the trained location were compared between the two subgroups categorized based on the baseline interhemispheric visual RSFC using the K-means^++^ clustering, using analysis of covariance models, with age, sex, and lesion volume in the visual cortex as covariates. A linear mixed-effects model for repeated measures was used to examine the fixed effects of the subgroups (high vs. low visual RSFC subgroups), time (baseline vs. 2 month-follow-up), and their interaction with the MTD score in the trained location. A random subject effect, age, sex, lesion volume in the visual cortex, and baseline MTD score were included also into the model

All the statistical tests were two-tailed, and the statistical threshold was set at *p* < 0.05. All statistical analysis was performed using the Stata SE (Stata Corp, College Station, TX, USA). The Bonferroni-corrected threshold of *p* < 0.0016 (0.05/3) was also used for three comparisons of the ipsilesional, interhemispheric, and contralesional visual cortex.

**Supplementary Results**

***Neurobehavioral associations in the control group***

In the control group, the MTD score in the affected hemi-field was not significantly associated with the mean RSFC of the ipsilesional and contralesional visual regions before and after the 2-month control period (ipsilesional visual RSFC: pre-control period, *β* = -0.095, *p* = 0.753: post-control period, *β* = 0.113, *p* = 0.706; contralesional visual RSFC: pre-control period, *β* = 0.205, *p* = 0.463: post-control period, *β* = 0.324, *p* = 0.260; Pearson correlation analysis). The MTD score in the affected hemi-field showed a positive association with the mean RSFC of the interhemispheric visual regions only after the 2-month control period , but was not significant after the Bonferroni-correction (interhemispheric visual RSFC: pre-control period, *β* = 0.105, *p* = 0.769: post-control period, *β* = 0.677, Bonferroni-uncorrected *p* = 0.048).

***Predictive brain biomarker in the control group***

In the control group, the baseline mean RSFC of the ipsilesional, interhemispheric, and contralesional visual regions was not associated with the MTD score in the affected hemi-field after the 2-month control period (ipsilesional, *β* = -0.195, *p* = 0.496; interhemispheric, *β* = -0.016, *p* = 0.964; contralesional, *β* = 0.108, *p* = 0.691; Pearson correlation analysis).

***Subgroup analysis results in the control group***

In the control group, the baseline MTD score in the affected hemi-field showed non-significant differences between the high (n = 6) and low (n = 9) visual RSFC subgroups (*p* = 0.323; analysis of covariance), categorized using the K-means^++^ clustering based on the baseline interhemispheric visual RSFC. The interaction between time (baseline vs. 2-month follow-up) and the visual RSFC subgroups (high vs. low) in the control group was not significant on the MTD score in the affected hemi-field (*z* = -0.960, *p* for interaction = 0.339; a linear mixed-effects model for repeated measures).

Regarding visual RSFC characteristics at baseline, enhanced RSFCs were observed in the interhemispheric (Bonferroni-uncorrected *p* = 0.007; analysis of covariance) visual regions, but not in the ipsilesional (*p* = 0.055) and contralesional (*p* = 0.160) regions of the high visual RSFC subgroup compared to the low visual RSFC subgroup. The interactions between time (baseline vs. 2-month follow-up) and the visual RSFC subgroups in the control group (high vs. low) were not significant on the ipsilesional (*z* = -0.76, *p* for interaction = 0.445; a linear mixed-effects model for repeated measures), interhemispheric (*z* = -0.96, *p* for interaction = 0.339), and the contralesional visual RSFCs (*z* = 0.12, *p* for interaction = 0.905).

***Neural plasticity in the control group***

Regarding the 2-month changes in the control group, changes in the ipsilesional, interhemispheric, and contralesional visual RSFC were not associated with change in the MTD score of the affected hemi-field (ipsilesional, *r* = -0.374, *p* = 0.260; interhemispheric, *r* = 0.126, *p* = 0.696; contralesional, *r* = 0.318, *p* = 0.360; Spearman correlation analysis).

***Baseline brain-behavior relationships with baseline VFD severity, patient age, time since stroke, and lesion volume***

At baseline in the VPL training group (n = 31), the whole lesion volume was negatively associated with the MTD score in the affected hemi-field (*r* = -0.579, Bonferroni-corrected *p* < 0.001; Spearman correlation analysis) and the interhemispheric visual RSFC (*r* = -0.527, Bonferroni-uncorrected *p* = 0.003; Spearman correlation analysis). Additionally, lesion volume in the visual cortex was negatively associated with the MTD score in the affected hemi-field (*r* = -0.611, Bonferroni-corrected *p* < 0.001; Spearman correlation analysis), the ipsilesional visual RSFC (*r* = -0.430, Bonferroni-uncorrected *p* = 0.016; Spearman correlation analysis), and the interhemispheric visual RSFC (*r* = -0.576, Bonferroni-corrected *p* < 0.001; Spearman correlation analysis).

At baseline, the whole lesion volume was not associated with the ipsilesional and contralesional visual RSFC (ipsilesional, *r* = -0.346, *p* = 0.057; contralesional, *r* = -0.045, *p* = 0.808; Spearman correlation analysis). The lesion volume in the visual cortex were not associated with the contralesional visual RSFC (*r* = -0.093, *p* = 0.616; Spearman correlation analysis). In addition, time since stroke onset and patient age were not associated with the MTD score in the affected hemi-field and the ipsilesional, interhemispheric, and contralesional visual RSFCs at baseline in the training group.

At baseline, MTD score in the affected hemifield was positively associated with the ipsilesional (*r* = 0.445, Bonferroni-uncorrected *p* = 0.013; Spearman correlation analysis) and interhemispheric (*r* = 0.627, Bonferroni-corrected *p* < 0.001; Spearman correlation analysis) visual RSFC at baseline in the training group.

***Auxiliary analysis for baseline predictive factors of VPL-induced changes in MTD scores***

In the multivariate stepwise regression analysis including all factors with backward selection, the baseline interhemispheric visual RSFC (*p* = 0.158; multivariate stepwise regression analysis) was included at the significance level of 0.20, but was not significantly correlated with VPL-induced MTD changes in the training group (n = 31). Other factors were eliminated from the model (*p* > 0.20): baseline contralesional visual RSFC, *p* = 0.974; patient age, *p* = 0.838; whole brain lesion volume, *p* = 0.774; lesion volume in the visual cortex, *p* = 0.894; baseline ipsilesional visual RSFC, *p* = 0.722; time since stroke, *p* = 0.438; baseline MTD in the affected hemifield, *p* = 0.210; multivariate stepwise regression analysis).

In the control group (n = 15), the baseline MTD scores of affected hemi-field (*p* = 0.070), baseline interhemispheric visual RSFC (*p* = 0.109), and lesion volume in the visual cortex (*p* = 0.119) were included at the significance level of 0.20, but none of these factors were significantly correlated with MTD changes during the 2-month control period, using the multivariate stepwise regression analysis.

***Auxiliary subgroup analyses: Effects of baseline MTD and lesion volume in VPL-induced VFD recovery***

Baseline MTD score in the affected hemi-field showed significant differences between the two subgroups (Bonferroni-uncorrected *p* = 0.003; analysis of covariance), categorized using the K-means^++^ clustering based on the baseline MTD scores (silhouette coefficient = 0.795). The interaction between time (pre-VPL vs. post-VPL) and the baseline subgroups according to MTD (high vs. low) (*z* = 0.55, *p* for interaction = 0.579; a linear mixed-effects model for repeated measures), along with the time effect (*z* = 0.59, *p* = 0.552), was not significant on changes in the MTD score in the affected hemi-field. The subgroup effect (*z* = 3.47, *p* = 0.001) was significant on changes in the MTD score in the affected hemi-field. The MTD score of the affected hemi-field showed nonsignificant changes after VPL both in the high subgroup (n = 19, *p* = 0.204; paired t-test) and the low subgroup (n = 12, *p* = 0.293).

Baseline MTD score in the affected hemi-field showed significant differences between the two subgroups (Bonferroni-uncorrected *p* = 0.006; analysis of covariance), categorized using the K-means^++^ clustering based on the baseline lesion volume in the visual cortex (silhouette coefficient = 0.715). The interaction between time (pre-VPL vs. post-VPL) and the baseline subgroups according the lesion volume in the visual cortex (high vs. low) (*z* = -0.01, *p* for interaction = 0.995; a linear mixed-effects model for repeated measures), along with the subgroup effect (*z* = 0.21, *p* = 0.831) and the time effect (*z* = 1.00, *p* = 0.316), was not significant on changes in the MTD score in the affected hemi-field. The MTD score of the affected hemi-field showed nonsignificant changes after VPL both in the high subgroup (n = 20, *p* = 0.281; paired t-test) and the low subgroup (n = 11, *p* = 0.158).

Baseline MTD score in the affected hemi-field showed significant differences between the two subgroups (Bonferroni-uncorrected *p* = 0.005; analysis of covariance), categorized using the K-means^++^ clustering based on the baseline whole brain lesion volume (silhouette coefficient = 0.736). The interaction between time (pre-VPL vs. post-VPL) and the baseline subgroups according the whole brain lesion volume (high vs. low) (*z* = 0.01, *p* for interaction = 0.990; a linear mixed-effects model for repeated measures), along with the subgroup effect (*z* = 0.27, *p* = 0.790) and the time effect (*z* = 0.94, *p* = 0.347), was not significant on changes in the MTD score in the affected hemi-field. The MTD score of the affected hemi-field showed nonsignificant changes after VPL both in the high subgroup (n = 21, *p* = 0.254; paired t-test) and the low subgroup (n = 10, *p* = 0.210).

***Sensitivity analyses: Effects of baseline whole brain lesion volume in VPL-induced VFD recovery***

By replacing lesion volume in the visual cortex with the whole brain volume as a covariate, the baseline interhemispheric visual RSFC remained the strong positive association with the post-VPL MTD score (ipsilesional, *β* = 0.412, Bonferroni-uncorrected *p* = 0.015; interhemispheric, *β* = 0.632, Bonferroni-corrected *p* < 0.001, contralesional, *β* = 0.395, Bonferroni-uncorrected *p* = 0.013; Pearson correlation analysis).

After replacing lesion volume in the visual cortex with the whole brain volume as a covariate, the interaction between time (pre-VPL vs. post-VPL) and the visual RSFC subgroups according to baseline interhemispheric visual RSFC (high vs. low) remained significant on changes in the MTD score in the affected hemi-field (*z* = 2.08, Bonferroni-uncorrected *p* for interaction = 0.038; a linear mixed-effects model for repeated measures). However, the subgroup effect according to baseline interhemispheric visual RSFC (high vs. low)(*z* = 0.70, *p* = 0.482) and the time effect (pre-VPL vs. post-VPL)(*z* = -0.14, *p* = 0.885) remained not significant on changes in the MTD score in the affected hemi-field.

After replacing lesion volume in the visual cortex with the whole brain volume as a covariate, VPL-induced change in the mean RSFC of the interhemispheric visual region remained a positive association with VPL-induced change in the MTD score of the affected hemifield (ipsilesional, *r* = -0.005, *p* = 0.978; interhemispheric, *r* = 0.411, Bonferroni-uncorrected *p* = 0.038; contralesional, *r* = 0.180, *p* = 0.358; Spearman correlation analysis).

***Sensitivity analyses: Effects of baseline MTD in VPL-induced VFD recovery***

With the baseline MTD score as an additional covariate, the correlation between the baseline interhemispheric visual RSFC and post-VPL MTD score became a nonsignificant trend toward a positive association (ipsilesional, *β* = 0.134, *p* = 0.139; interhemispheric, *β* = 0.199, *p* = 0.076, contralesional, *β* = 0.080, *p* = 0.364; Pearson correlation analysis).

With the baseline MTD score as an additional covariate, VPL-induced change in the mean RSFC of the interhemispheric visual region was positively associated with VPL-induced change in the MTD score of the affected hemi-field, after adjusting for age, sex, baseline lesion volume in the visual cortex, and baseline MTD score (ipsilesional, *r* = -0.019, *p* = 0.928; interhemispheric, *r* = 0.418, Bonferroni-uncorrected *p* = 0.044; contralesional, *r* = 0.178, *p* = 0.396; Spearman correlation analysis).

***Predictive brain biomarker and Neural Plasticity in relation to Trained Location***

As in the MTD score of the affected hemi-field, the mean RSFC of the interhemispheric visual regions at baseline showed the strongest positive association with the MTD score in the trained location following VPL, in the training group (ipsilesional, *β* = 0.482, Bonferroni-uncorrected *p* = 0.004; interhemispheric, *β* = 0.580, Bonferroni-uncorrected *p* = 0.002; contralesional, *β* = 0.341, Bonferroni-uncorrected *p* = 0.034; Pearson correlation analysis). Baseline MTD score in the trained location did not significantly differ between the two subgroups (*p* = 0.065; analysis of covariance), categorized using the K-means^++^ clustering based on the baseline interhemispheric visual RSFC. The interaction between time (pre-VPL vs. post-VPL) and the baseline subgroups according the interhemispheric visual RSFC (high vs. low) (*z* = 1.48, *p* for interaction = 0.139; a linear mixed-effects model for repeated measures), along with the subgroup effect (*z* = 0.21, *p* = 0.838) and the time effect (*z* = 0.10, *p* = 0.920), was not significant on changes in the MTD score in the trained location.

In the training group, VPL-induced changes in the mean RSFC of the ipsilesional, interhemispheric, and contralesional visual regions were not associated with VPL-induced change in the MTD score of the trained location, after adjusting for age, sex, and lesion volume in the visual cortex (ipsilesional, *r* = -0.070, *p* = 0.711; interhemispheric, *r* = 0.352, *p* = 0.065; contralesional, *r* = 0.081, *p* = 0.664; Spearman correlation analysis).

**Supplementary References**

1. Lee, E.J. *et al.* Digital Therapeutics With Visual Discrimination Training for Cortical Blindness in Patients With Chronic Stroke. *J. Stroke* **25**, 409-412 (2023).

2. Jenkinson M, Beckmann C.F, Behrens T.E., Woolrich M.W., Smith S.M. Fsl. *Neuroimage* **62,** 782-790 (2012).

3. Pustina D, *et al.* Automated segmentation of chronic stroke lesions using LINDA: Lesion identification with neighborhood data analysis. *Hum. Brain Mapp.* **37,** 1405-1421 (2016).

4. Behzadi, Y., Restom, K., Liau, J. & Liu, T. T. A component based noise correction method (CompCor) for BOLD and perfusion based fMRI. *Neuroimage* 37, 90-101 (2007).

5. Desikan R.S*, et al.* An automated labeling system for subdividing the human cerebral cortex on MRI scans into gyral based regions of interest. *Neuroimage* **31**, 968-980 (2006).

6. Liu C. F., *et al.* Digital 3D Brain MRI Arterial Territories Atlas. *Sci. Data* **10,** 74 (2023).

**Supplementary Figures and Tables**

| 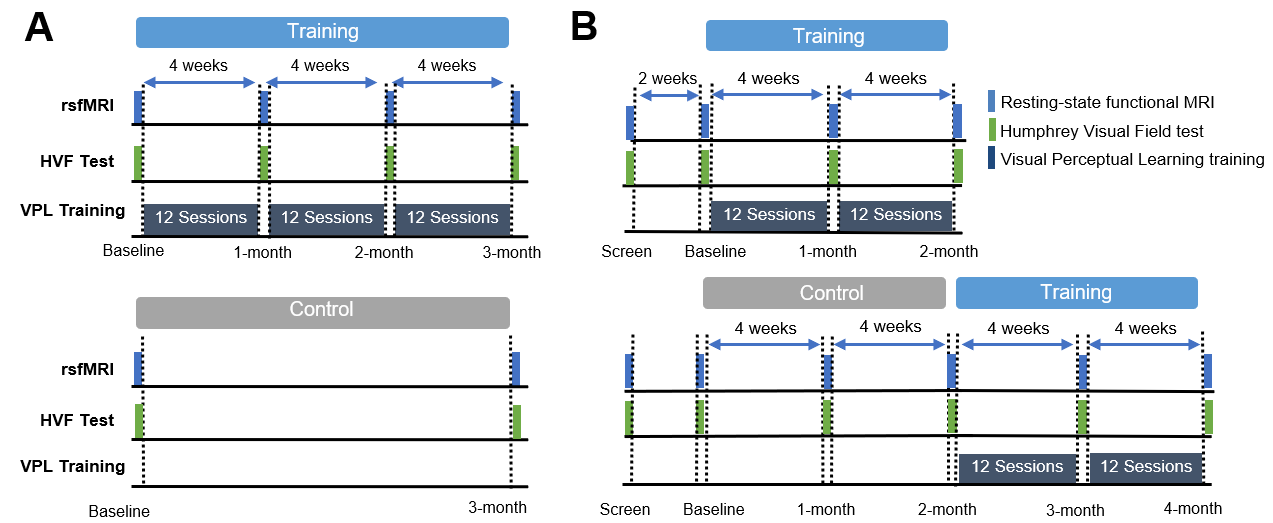 |
| --- |
| **Supplementary Figure 1.** Previous two study designs used in retrospective analysis  **(A)** Our retrospective analysis included patients with VFD following chronic ischemic stroke who received 24 sessions of VPL training for 2 months as the VPL training group from our previous first study^1^.  **(B)** Our retrospective analysis included the patients who received 24 sessions of VPL training for 2 months (VPL training group) and the patients who received no-training for 2 months (control group) from our previous second study.  Abbreviations: HVF, Humphrey Visual Field Test; rsfMRI, resting-state functional magnetic resonance imaging; MRI, magnetic resonance imaging; VPL, visual perceptual learning |

| 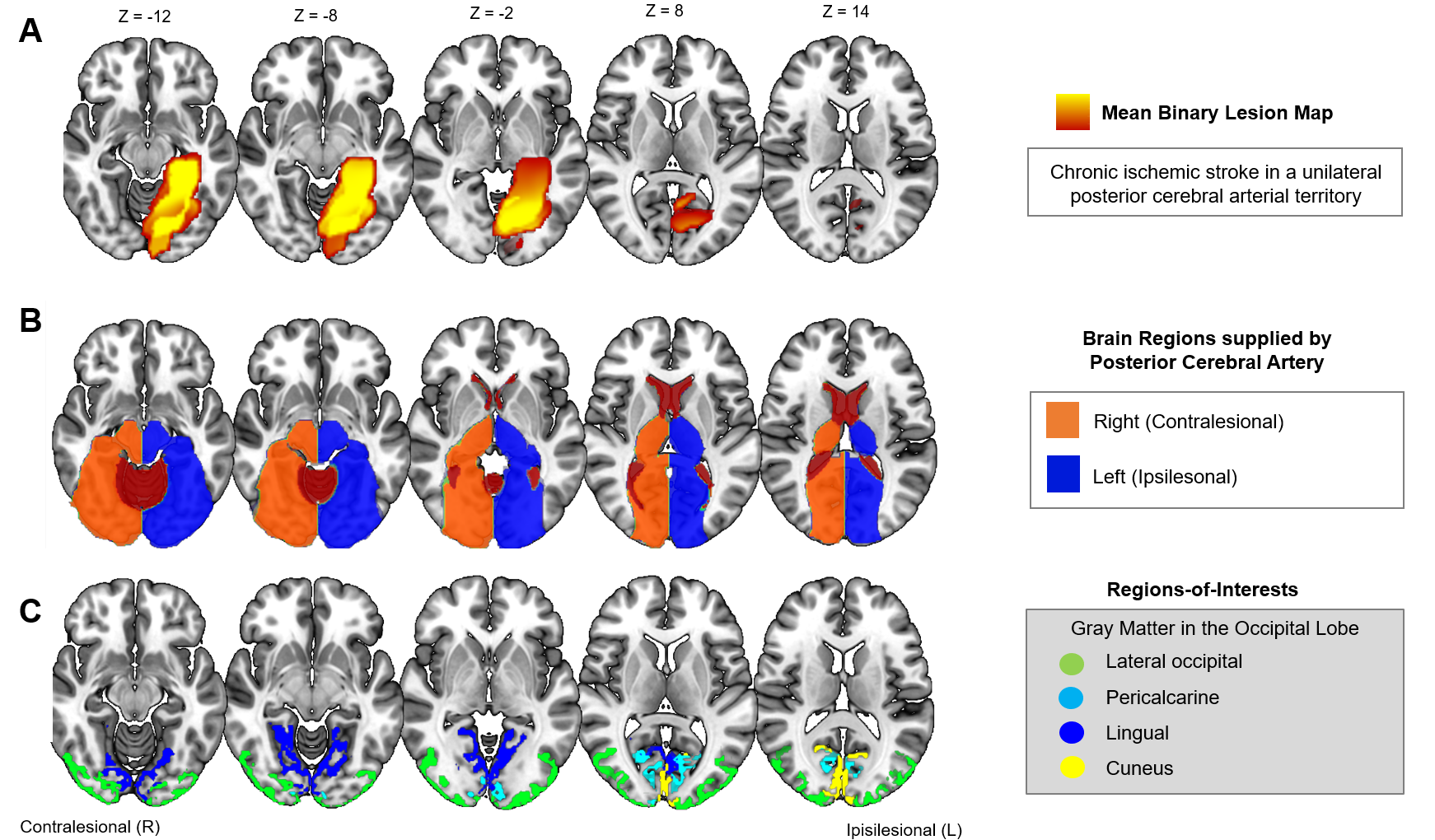 |
| --- |
| **Supplementary Figure 2.** A schematic figure representing lesion location, regions supplied by PCA, and regions included in the ROIs.  **(A)** The binary lesion map (red-to-yellow) averaged for our participants is overlaid on the brain MNI template  **(B)** The brain regions (orange, right side; blue, left side) supplied by posterior cerebral artery are indicated based on the Digital 3D Brain MRI Arterial Territories Atlas^6^.  **(C)** Our regions-of-interests include gray matter regions of the cuneus, pericalcarine, lingual, and lateral occipital cortex in the occipital lobe, parcellated based on the Desikan-Killiany Atlas^5^.  Abbreviations: L, left; Montreal Neurological Institute; PCA, posterior cerebral artery; R, right |

**Supplementary Table 1**. Associations between visual RSFC and MTD score in the affected hemi-field at pre- and post- VPL

|  | Brain-behavior association  at pre-VPL | | Brain-behavior association at post-VPL | |
| --- | --- | --- | --- | --- |
| Visual RSFC | *β* | *p* | *β* | *p* |
| Ipsilesional |  |  |  |  |
| IL LatOC - IL CUN | 0.073 | 0.645 | 0.276 | 0.096 |
| IL LatOC - IL PCAL | 0.418 | 0.005* | 0.255 | 0.146 |
| IL LatOC - IL LING | 0.331 | 0.051 | 0.196 | 0.221 |
| IL CUN - IL PCAL | 0.083 | 0.623 | 0.205 | 0.289 |
| IL CUN - IL LING | 0.162 | 0.322 | 0.313 | 0.160 |
| IL PCAL - IL LIN | 0.248 | 0.165 | 0.235 | 0.227 |
| Between hemispheres |  |  |  |  |
| IL LatOC - CL LatOC | 0.550 | <0.001* | 0.456 | 0.018 |
| IL LatOC - CL CUN | -0.006 | 0.971 | 0.293 | 0.061 |
| IL LatOC - CL PCAL | 0.070 | 0.671 | 0.309 | 0.053 |
| IL LatOC - CL LING | 0.202 | 0.224 | 0.142 | 0.379 |
| IL CUN - CL LatOC | 0.075 | 0.639 | 0.244 | 0.144 |
| IL CUN - CL CUN | 0.291 | 0.125 | 0.364 | 0.086 |
| IL CUN - CL PCAL | 0.368 | 0.042 | 0.337 | 0.082 |
| IL CUN - CL LING | 0.302 | 0.066 | 0.195 | 0.301 |
| IL PCAL - CL LatOC | 0.461 | 0.003* | 0.294 | 0.108 |
| IL PCAL - CL CUN | 0.273 | 0.146 | 0.504 | 0.009 |
| IL PCAL - CL PCAL | 0.437 | 0.028 | 0.645 | 0.001* |
| IL PCAL - CL LING | 0.440 | 0.012 | 0.518 | 0.008 |
| IL LIN - CL LatOC | 0.313 | 0.070 | 0.232 | 0.153 |
| IL LIN - CL CUN | 0.307 | 0.056 | 0.721 | <0.001* |
| IL LIN - CL PCAL | 0.374 | 0.041 | 0.531 | 0.005 |
| IL LIN - CL LIN | 0.488 | 0.011 | 0.603 | 0.002* |
| Contralesional |  |  |  |  |
| CL LatOC - CL CUN | -0.021 | 0.893 | 0.183 | 0.276 |
| CL LatOC - CL PCAL | 0.149 | 0.368 | -0.043 | 0.791 |
| CL LatOC - CL LING | 0.191 | 0.230 | -0.096 | 0.566 |
| CL CUN - CL PCAL | 0.352 | 0.022 | 0.234 | 0.141 |
| CL CUN - CL LING | 0.286 | 0.067 | 0.315 | 0.052 |
| CL PCAL - CL LIN | 0.266 | 0.090 | 0.052 | 0.755 |

In the VPL training group, associations between visual RSFC and MTD score in the affected hemi-field were examined with age, sex, and lesion volume in the visual cortex as covariates at pre- and post- VPL training.

Abbreviations: IL, ipsilesional; CL, contralesional; CUN, Cuneus cortex; LatOC, Lateral occipital cortex; LING, Lingual gyrus; MTD, mean total deviation; PCAL, Pericalcarine cortex; RSFC, resting-state functional connectivity; VPL, visual perceptual learning

**p* < 0.0083 (0.05/6) for the ipsilesional and contralesional visual RSFCs; *p* < 0.0031 (0.05/16) for interhemispheric visual RSFC

**Supplementary Table 2**. Associations between visual RSFC at baseline and MTD score of the affected hemi-field at post-VPL

|  | Association with post-VPL MTD score of the affected hemi-field | |
| --- | --- | --- |
| Baseline Visual RSFC | *β* | *p* |
| Ipsilesional |  |  |
| IL LatOC - IL CUN | 0.126 | 0.436 |
| IL LatOC - IL PCAL | 0.416 | 0.007* |
| IL LatOC - IL LING | 0.346 | 0.046 |
| IL CUN - IL PCAL | 0.147 | 0.392 |
| IL CUN - IL LING | 0.249 | 0.132 |
| IL PCAL - IL LIN | 0.126 | 0.022 |
| Between hemispheres |  |  |
| IL LatOC - CL LatOC | 0.482 | 0.004 |
| IL LatOC - CL CUN | 0.034 | 0.840 |
| IL LatOC - CL PCAL | 0.190 | 0.253 |
| IL LatOC - CL LING | 0.271 | 0.107 |
| IL CUN - CL LatOC | 0.114 | 0.487 |
| IL CUN - CL CUN | 0.389 | 0.042 |
| IL CUN - CL PCAL | 0.352 | 0.059 |
| IL CUN - CL LING | 0.361 | 0.030 |
| IL PCAL - CL LatOC | 0.419 | 0.010 |
| IL PCAL - CL CUN | 0.334 | 0.080 |
| IL PCAL - CL PCAL | 0.568 | 0.004 |
| IL PCAL - CL LING | 0.482 | 0.007 |
| IL LIN - CL LatOC | 0.327 | 0.064 |
| IL LIN - CL CUN | 0.367 | 0.024 |
| IL LIN - CL PCAL | 0.565 | 0.001* |
| IL LIN - CL LIN | 0.632 | 0.001* |
| Contralesional |  |  |
| CL LatOC - CL CUN | -0.032 | 0.842 |
| CL LatOC - CL PCAL | 0.259 | 0.122 |
| CL LatOC - CL LING | 0.221 | 0.172 |
| CL CUN - CL PCAL | 0.285 | 0.077 |
| CL CUN - CL LING | 0.261 | 0.105 |
| CL PCAL - CL LIN | 0.350 | 0.026 |

In the VPL training group, associations between visual functional connectivity at baseline and MTD score in the affected hemi-field at post-VPL training were investigated using Pearson correlation analysis with age, sex, and lesion volume in the visual cortex as covariates.

Abbreviations: IL, ipsilesional; CL, contralesional; CUN, Cuneus cortex; LatOC, Lateral occipital cortex; LING, Lingual gyrus; MTD, mean total deviation; PCAL, Pericalcarine cortex; RSFC, resting-state functional connectivity; VPL, visual perceptual learning

**p* < 0.0083 (0.05/6) for the ipsilesional and contralesional visual RSFC; *p* < 0.0031 (0.05/16) for interhemispheric visual RSFC

**Supplementary Table 3**. Effects of baseline interhemispheric visual RSFC on the brain in relation to VPL training

|  | Low visual RSFC group | | High visual RSFC group | | *p* for baseline | *p* for subgroup x visit interaction |
| --- | --- | --- | --- | --- | --- | --- |
|  | (n = 16) | | (n = 15) | |  |  |
| Visual Functional Connectivity | Baseline | Post-VPL | Baseline | Post-VPL |  |  |
| Ipsilesional |  |  |  |  |  |  |
| IL LatOC - IL CUN | 0.201 (0.336) | 0.217 (0.295) | 0.419 (0.319) | 0.445 (0.371) | 0.002* | 0.927 |
| IL LatOC - IL PCAL | 0.186 (0.306) | 0.136 (0.339) | 0.496 (0.293) | 0.499 (0.456) | <0.001* | 0.690 |
| IL LatOC - IL LING | 0.315 (0.313) | 0.227 (0.363) | 0.676 (0.320) | 0.436 (0.295) | 0.002 | 0.199 |
| IL CUN - IL PCAL | 0.514 (0.484) | 0.668 (0.412) | 1.146 (0.248) | 1.143 (0.261) | <0.001* | 0.094 |
| IL CUN - IL LING | 0.296 (0.323) | 0.396 (0.341) | 0.975 (0.310) | 0.822 (0.354) | <0.001* | 0.024 |
| IL PCAL - IL LIN | 0.371 (0.283) | 0.574 (0.350) | 1.085 (0.242) | 0.928 (0.383) | <0.001* | <0.001* |
| Between hemispheres |  |  |  |  |  |  |
| IL LatOC - CL LatOC | 0.944 (0.329) | 0.822 (0.409) | 1.320 (0.294) | 1.166 (0.368) | 0.002* | 0.819 |
| IL LatOC - CL CUN | 0.258 (0.340) | 0.251 (0.263) | 0.407 (0.282) | 0.335 (0.353) | 0.0034 | 0.628 |
| IL LatOC - CL PCAL | 0.411 (0.351) | 0.277 (0.2630 | 0.545 (0.320) | 0.433 (0.363) | 0.041 | 0.869 |
| IL LatOC - CL LING | 0.433 (0.201) | 0.365 (0.288) | 0.720 (0.253) | 0.458 (0.398) | <0.001* | 0.166 |
| IL CUN - CL LatOC | 0.167 (0.346) | 0.121 (0.400) | 0.384 (0.262) | 0.392 (0.335) | 0.022 | 0.597 |
| IL CUN - CL CUN | 0.658 (0.439) | 0.705 (0.306) | 1.250 (0.308) | 1.243 (0.384) | <0.001* | 0.685 |
| IL CUN - CL PCAL | 0.349 (0.361) | 0.504 (0.5160 | 0.920 (0.238) | 0.986 (0.312) | <0.001* | 0.486 |
| IL CUN - CL LING | 0.319 (0.191) | 0.356 (0.432) | 0.912 (0.295) | 0.939 (0.352) | <0.001* | 0.941 |
| IL PCAL - CL LatOC | 0.082 (0.326) | 0.038 (0.422) | 0.460 (0.245) | 0.401 (0.343) | <0.001* | 0.893 |
| IL PCAL - CL CUN | 0.155 (0.415) | 0.263 (0.293) | 0.958 (0.219) | 0.940 (0.302) | <0.001* | 0.249 |
| IL PCAL - CL PCAL | 0.237 (0.330) | 0.308 (0.396) | 1.113 (0.258) | 1.027 (0.350) | <0.001* | 0.082 |
| IL PCAL - CL LING | 0.138 (0.274) | 0.162 (0.417) | 0.942 (0.295) | 0.888 (0.347) | <0.001* | 0.489 |
| IL LIN - CL LatOC | 0.270 (0.288) | 0.106 (0.395) | 0.578 (0.335) | 0.416 (0.310) | 0.020 | 0.988 |
| IL LIN - CL CUN | 0.264 (0.222) | 0.297 (0.337) | 0.864 (0.293) | 0.751 (0.264) | <0.001* | 0.112 |
| IL LIN - CL PCAL | 0.285 (0.175) | 0.309 (0.362) | 0.927 (0.277) | 0.833 (0.370) | <0.001* | 0.333 |
| IL LIN - CL LIN | 0.387 (0.251) | 0.393 (0.395) | 1.157 (0.368) | 1.100 (0.472) | <0.001* | 0.558 |
| Contralesional |  |  |  |  |  |  |
| CL LatOC - CL CUN | 0.347 (0.391) | 0.314 (0.336) | 0.388 (0.189) | 0.408 (0.351) | 0.533 | 0.682 |
| CL LatOC - CL PCAL | 0.498 (0.414) | 0.494 (0.243) | 0.558 (0.267) | 0.484 (0.336) | 0.358 | 0.532 |
| CL LatOC - CL LING | 0.571 (0.193) | 0.564 (0.313) | 0.706 (0.308) | 0.559 (0.363) | 0.153 | 0.241 |
| CL CUN - CL PCAL | 0.824 (0.424) | 1.024 (0.420) | 1.140 (0.301) | 1.192 (0.280) | 0.001* | 0.331 |
| CL CUN - CL LING | 0.656 (0.381) | 0.743 (0.393) | 0.950 (0.267) | 1.071 (0.208) | 0.001* | 0.795 |
| CL PCAL - CL LIN | 0.764 (0.393) | 0.964 (0.347) | 1.263 (0.320) | 0.408 (0.241) | <0.001* | 0.025 |

In the VPL training group, baseline between-subgroup differences in visual RSFC were examined using analysis of covariance models with age, sex, and lesion volume in the visual cortex as covariates. A mixed-effects model repeated-measures analysis were used to examine subgroup effects of baseline interhemispheric visual functional connectivity on visual RSFCs in relation to VPL. Subgroup (high vs. low visual RSFC group), visit (pre- vs. post- VPL training), and subgroup-by-visit interaction were included as fixed effects, while the within-subjects factor was included as a random effect. Age, sex, and lesion volume in the visual cortex were included as covariates.

Abbreviations: IL, ipsilesional; CL, contralesional; CUN, Cuneus cortex; LatOC, Lateral occipital cortex; LING, Lingual gyrus; MTD, mean total deviation; PCAL, Pericalcarine cortex; RSFC, resting-state functional connectivity; VPL, visual perceptual learning

**p* < 0.0083 (0.05/6) for the ipsilesional and contralesional visual RSFC; *p* < 0.0031 (0.05/16) for the interhemispheric visual RSFC

**Supplementary Table 4**. Associations between VPL-induced changes in visual RSFC and in MTD score of the affected hemi-field

|  | Association with change in MTD score of the affected hemi-field | |
| --- | --- | --- |
| Change in visual RSFC | *β* | *p* |
| Ipsilesional |  |  |
| IL LatOC - IL CUN | 0.188 | 0.375 |
| IL LatOC - IL PCAL | 0.151 | 0.465 |
| IL LatOC - IL LING | -0.003 | 0.967 |
| IL CUN - IL PCAL | -0.130 | 0.517 |
| IL CUN - IL LING | -0.126 | 0.501 |
| IL PCAL - IL LIN | -0.163 | 0.400 |
| Between hemispheres |  |  |
| IL LatOC - CL LatOC | 0.437 | 0.036 |
| IL LatOC - CL CUN | 0.237 | 0.235 |
| IL LatOC - CL PCAL | 0.063 | 0.770 |
| IL LatOC - CL LING | 0.081 | 0.691 |
| IL CUN - CL LatOC | 0.447 | 0.031 |
| IL CUN - CL CUN | 0.113 | 0.538 |
| IL CUN - CL PCAL | 0.189 | 0.313 |
| IL CUN - CL LING | -0.007 | 0.984 |
| IL PCAL - CL LatOC | 0.315 | 0.115 |
| IL PCAL - CL CUN | 0.219 | 0.283 |
| IL PCAL - CL PCAL | 0.201 | 0.327 |
| IL PCAL - CL LING | 0.127 | 0.547 |
| IL LIN - CL LatOC | 0.199 | 0.318 |
| IL LIN - CL CUN | 0.247 | 0.184 |
| IL LIN - CL PCAL | 0.052 | 0.791 |
| IL LIN - CL LIN | 0.437 | 0.232 |
| Contralesional |  |  |
| CL LatOC - CL CUN | 0.385 | 0.041 |
| CL LatOC - CL PCAL | -0.217 | 0.276 |
| CL LatOC - CL LING | -0.074 | 0.686 |
| CL CUN - CL PCAL | 0.284 | 0.132 |
| CL CUN - CL LING | 0.240 | 0.212 |
| CL PCAL - CL LIN | -0.185 | 0.346 |

In the VPL training group, associations between VPL-induced changes in visual RSFC and in MTD score of the affected hemi-field were investigated using Pearson correlation analysis with age, sex, and lesion volume in the visual cortex as covariates.

Abbreviations: IL, ipsilesional; CL, contralesional; CUN, Cuneus cortex; LatOC, Lateral occipital cortex; LING, Lingual gyrus; MTD, mean total deviation; PCAL, Pericalcarine cortex; RSFC, resting-state functional connectivity; VPL, visual perceptual learning

**p* < 0.0083 (0.05/6) for the ipsilesional and contralesional visual RSFCs; *p* < 0.0031 (0.05/16) for the interhemispheric visual RSFC

**Supplementary Table 5**. Demographic and clinical characteristics of the study participants

|  | VPL training group | | | Control group | | |
| --- | --- | --- | --- | --- | --- | --- |
|  | Low visual RSFC subgroup | High visual RSFC subgroup | *p* | Low visual RSFC subgroup | High visual RSFC subgroup | *p* |
|  | (n = 16) | (n = 15) |  | (n = 9) | (n = 6) |  |
| Age | 59.9 (16.1) | 54.8 (16.0) | 0.379 | 48.6 (17.4) | 53.7 (16.6) | 0.580 |
| Male | 13 (81.3) | 13 (86.7) | 0.682 | 8 (88.9) | 6 (100) | 0.398 |
| Infarct in the left hemisphere (%) | 6 (37.5) | 6 (40.0) | 0.886 | 3 (33.3) | 4 (66.7) | 0.205 |
| Hemianopia: quadrantanopia (%) | 9:7 (56.3:43.8) | 4:11 (26.7:73.3) | 0.095 | 6:3 (66.7:33.3) | 2:4 (33.3:66.7) | 0.205 |
| Duration since stroke onset (months) | 32.9 (32.5) | 25.8 (29.7) | 0.533 | 21.6 (32.2) | 10.5 (2.67) | 0.423 |
| Whole brain stroke lesion volume (mm^3^) | 33.8 (29.6) | 14.4 (14.7) | 0.029* | 45.1 (30.3) | 12.7 (7.88) | 0.025* |
| Lesion volume in the visual cortex (mm^3^) | 19.7 (15.0) | 8.18 (8.42) | 0.014* | 27.7 (15.5) | 6.71 (4.07) | 0.007* |

Baseline between-subgroup differences in demographic and clinical characteristics were examined in the VPL training and control groups using an independent t-test or chi-squared test. The continuous variables are indicated as mean (standard deviation), while the categorical variables are indicated as number (%)

Abbreviations: RSFC, resting-state functional connectivity; VPL, visual perceptual learning

**p* < 0.05
